# The staircase cluster randomised trial design: A pragmatic alternative to the stepped wedge

**DOI:** 10.1177/09622802231202364

**Published:** 2023-11-30

**Authors:** Kelsey L Grantham, Andrew B Forbes, Richard Hooper, Jessica Kasza

**Affiliations:** 1 School of Public Health and Preventive Medicine, Monash University, Melbourne, Australia; 2 Wolfson Institute of Population Health, 4617 Queen Mary University of London, London, UK

**Keywords:** Clinical trial design, cluster randomised trial, incomplete design, intracluster correlation, sample size

## Abstract

This article introduces the ‘staircase’ design, derived from the zigzag pattern of steps along the diagonal of a stepped wedge design schematic where clusters switch from control to intervention conditions. Unlike a complete stepped wedge design where all participating clusters must collect and provide data for the entire trial duration, clusters in a staircase design are only required to be involved and collect data for a limited number of pre- and post-switch periods. This could alleviate some of the burden on participating clusters, encouraging involvement in the trial and reducing the likelihood of attrition. Staircase designs are already being implemented, although in the absence of a dedicated methodology, approaches to sample size and power calculations have been inconsistent. We provide expressions for the variance of the treatment effect estimator when a linear mixed model for an outcome is assumed for the analysis of staircase designs in order to enable appropriate sample size and power calculations. These include explicit variance expressions for basic staircase designs with one pre- and one post-switch measurement period. We show how the variance of the treatment effect estimator is related to key design parameters and demonstrate power calculations for examples based on a real trial.

## 1. Introduction

Longitudinal cluster randomised trial designs such as the stepped wedge are made up of sequences where clusters may switch between implementing the control and intervention conditions over several trial periods.^1^ The standard stepped wedge design, where all clusters begin in the control condition and then switch over just once to the intervention condition at staggered times over the trial, requires clusters to collect and provide measurements on subjects’ outcomes in every period of the trial. In some trial settings, there may be plenty of time to stagger the introduction of the intervention in different clusters, but it may be burdensome or costly to collect individual-level data in each cluster for the entire trial duration. Indeed, trials in practice are implementing non-standard stepped wedge designs despite the absence of appropriate methodology. Pragmatic alternatives to the stepped wedge design that do not require clusters to be involved for the entire trial duration are urgently needed, together with the underlying statistical theory to support their implementation.

Derived from the zigzag pattern of steps along the main diagonal of a stepped wedge which has been shown to contain a large amount of information for estimation of the treatment effect,^2^ a staircase design is a novel longitudinal cluster randomised trial design in which the sequences consist of a *limited* number of pre-switch control periods and post-switch intervention periods. The staircase design shares several practical advantages with a stepped wedge design while reducing the implementation and data collection requirements from each cluster. As with a stepped wedge design, each sequence in a staircase design contains one unidirectional switch from the control to the intervention condition. This makes it suitable for testing interventions that cannot easily be revoked once implemented, such as education and training programmes where new knowledge is provided as part of the intervention. Moreover, this means that all participating clusters eventually receive the intervention during the course of the trial. Staircase designs also have the same staggered rollout of the intervention across clusters as with the stepped wedge, making it more logistically feasible to introduce the intervention over time. However, staircase designs may be more appealing to participating clusters than the stepped wedge: Each cluster receives the intervention sooner upon commencing data collection, and is only required to contribute data in a limited number of periods rather than in all periods of the trial.

Trials with staircase-like designs are already being implemented, driven by the need for a less burdensome design than the complete stepped wedge design. A trial from 2020, for example, sought to test the effectiveness of a peripheral intravenous catheter flushing education programme on all-cause catheter failure across several wards in a hospital.^3^ The researchers wanted a design that would enable the staggered rollout of the intervention, but with a limited number of periods in each sequence to minimise the measurement burden on the wards. Another trial with a limited number of pre- and post-switch measurement periods in each sequence sought to test whether an education programme to improve self-regulation could reduce students’ disruptive behaviour across schools in remote Aboriginal communities.^4^ The outcome measures for each student were determined by questionnaires completed by teachers and parents, and so data collection was somewhat onerous. The researchers stated that ‘the burden of data collection would have been too great in a stepped wedge’; in addition, a stepped wedge would have been too costly and also difficult to implement geographically as it would have required repeated physical collection of questionnaires from remote locations over an extended period of time.

While many of the trials with staircase-like designs have been referred to as stepped wedge designs,^4–7^ much of the methodology for stepped wedge designs assumes a complete design where all clusters provide measurements on subjects’ outcomes in all periods of the trial. Many formulae and publicly available tools for sample size and power calculations appropriate for stepped wedge designs do not readily extend to these types of ‘incomplete’ designs with periods of no measurement. This leads researchers into dangerous and uncharted territory at the trial design stage: formulae appropriate for stepped wedge designs may underestimate the required number of clusters for a desired level of power or overestimate trial power for a given sample size if applied to staircase designs. Despite some researchers explicitly acknowledging this issue,^4^ in many cases it remains unclear how researchers conducted sample size and power calculations for trials with a staircase design in the absence of dedicated methodology.

Staircase designs have so far only made peripheral appearances in methodology papers in the cluster randomised trial design literature. These types of designs have been used as examples of incomplete stepped wedge designs, most commonly a staircase design with one control period followed by two intervention periods in each sequence.^8,9^ Designs with measurements concentrated along the main zigzag diagonal of a stepped wedge design have also arisen from investigations of the efficiency of potentially incomplete stepped wedge designs.^10,11^ A staircase design with treatment sequences consisting of just one control period followed by one intervention period can also be viewed as an extension of the dog-leg design.^12^ While there is some indication that staircase designs can be efficient alternatives to the stepped wedge,^11^ knowledge of the mathematical and statistical properties of these designs is lacking, thus limiting the uptake and proper implementation of such designs.

In this article, we formally introduce the staircase design by describing its properties and providing formulae to enable appropriate sample size and power calculations when a linear mixed model for an outcome is assumed. In Section 2 we present the notation, statistical model and an expression for the variance of the treatment effect estimator appropriate for general staircase designs. Section 3 focuses on basic staircase designs with one pre- and one post-switch measurement period in each sequence which permit explicit formulae for the variance of the treatment effect estimator. In Section 4 we demonstrate sample size and power calculations for staircase designs motivated by a real trial example and describe and demonstrate the use of some publicly available tools appropriate for staircase designs. Section 5 offers a discussion of our results and describes areas for further research.

## 2. Staircase designs

### 2.1. Design characteristics

**Figure 1.**
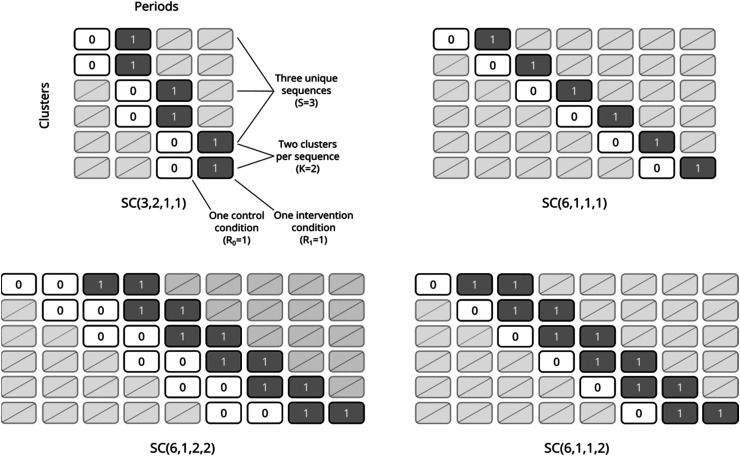
Design schematics for several staircase designs with 6 clusters: a basic staircase with two clusters assigned to each of three unique sequences (top left), a basic staircase with one cluster assigned to each of six unique sequences (top right), a balanced staircase with two control periods followed by two intervention periods in each sequence and one cluster assigned to each of six unique sequences (bottom left), and an imbalanced staircase with one control period followed by two intervention periods in each sequence and one cluster assigned to each of six unique sequences (bottom right).

A staircase design consists of overlapping treatment sequences that start in the control condition for one or more periods followed by the intervention condition for one or more periods, with periods of no measurements at one or both ends; design schematics for several staircase designs, each with six clusters, are shown in Figure 1. Each unique sequence begins taking measurements in a different period of the trial. We denote the general staircase design by $SC(S,K,R_{0},R_{1})$ , where *S* is the number of unique treatment sequences, with *K* clusters assigned to each sequence and comprising $R_{0}$ control periods followed by $R_{1}$ intervention periods. The total number of clusters included in the trial is therefore equal to SK and the total number of periods the trial spans is equal to $S+R_{0}+R_{1}-1$ . Clusters assigned to sequence *s* are observed in periods *s* to $s+R_{0}+R_{1}-1$ only. Further extensions to this family of designs are possible, for example, each of the unique treatment sequences might be offset by more than one period relative to the previous sequence, but in this article, we limit attention to the framework specified.

We define a *balanced staircase* design as having an equal number of pre-switch control periods and post-switch intervention periods in each sequence so that $R_{0}=R_{1}$ . We further define a *basic staircase* design as a special case of the balanced staircase design, with sequences comprised of measurements in just one control period followed by one intervention period $\left(R_{0}=R_{1}=1\right)$ , denoted by SC(S,K,1,1) . An *imbalanced staircase* design may have different numbers of pre- and post-switch periods in each sequence so that $R_{0}\neq R_{1}$ . The basic staircase design is comprised of SK clusters and a total of S+1 periods. While the basic staircase design is embedded within a standard stepped wedge design with *S* unique sequences and *K* clusters per sequence spanning S+1 periods (i.e., with just one control period before the first intervention step and one intervention period after the last step), staircase designs with more than one pre- and/or post-switch measurement period are not entirely contained within a standard stepped wedge with the same number of sequences. Sections 2.2 and 2.3 present a statistical model and the associated expression for the variance of the treatment effect estimator for general $SC(S,K,R_{0},R_{1})$ designs, and Section 3 presents explicit expressions for the basic staircase design.

### 2.2. Statistical model for continuous outcomes

#### 2.2.1. Individual-level model

Letting $Y_{skti}$ represent the outcome for subject i=1,…,m in cluster k=1,…,K assigned to sequence s=1,…,S in period $t=s,\ldots ,s+R_{0}+R_{1}-1$ , we consider the following mixed effects model for a continuous outcome:

(1) $$\begin{array}{r} Y_{skti}=Z_{st}\beta +X_{st}\theta +CP_{skt}+\epsilon_{skti},\epsilon_{skti}∼N(0,{\sigma_{\epsilon }}^{2}),\\ CP_{sk}=(CP_{sks},\ldots ,CP_{sk,s+R_{0}+R_{1}-1}{)}^{T}∼N_{R_{0}+R_{1}}(0,V_{CP_{sk}}) \end{array}$$

where β is a p -dimensional column vector of fixed time effects and $Z_{st}$ is a p -dimensional row vector specifying the form for the fixed effects corresponding to period *t*. This dimension *p* depends on the time parameterisation adopted, and we discuss some common choices of time parameterisation, such as categorical and linear time effects, in Section 2.2.2. $X_{st}$ is the treatment indicator for sequence *s* in the period *t*, θ is the treatment effect, and $\epsilon_{skti}$ is the subject-level error term. Model (1) is appropriate for a design where each participant provides only one measurement, and a modification for cohort designs is presented in Section 2.2.5. The term $CP_{skt}$ is the random effect for cluster *k* assigned to sequence *s* in period *t* and $V_{CP_{sk}}$ is the covariance matrix of the cluster-period random effects across the $R_{0}+R_{1}$ periods of measurement. We assume that all clusters assigned to all sequences have random effects with an identical distribution, and so we let $V_{CP}$ be the covariance matrix for the cluster-period random effects. We describe some possible structures for $V_{CP}$ in Section 2.2.3.

#### 2.2.2. Time parameterisations

The effect of time is encoded by specifying a form for $Z_{st}$ and β . In models for standard stepped wedge designs, it has been shown that including linear or categorical fixed time effects does not result in a different form for the variance of the treatment effect estimator.^13^ Hence, when a model of the form of model (1) is assumed, sample size expressions and power calculations are unaffected by the choice of time parameterisation. As a result, much of the development of the theory for the standard stepped wedge has considered only categorical period effects. However, as we will see in Section 3, for designs such as the staircase the variance of the treatment effect estimator is not invariant to the selected time parameterisation, i.e., the form of the time effects will have an impact on the variance of the treatment effect estimator. In our development we consider both categorical period effects and linear time effects, which may be a more appropriate assumption in some settings.^14^ Categorical period effects are returned if $Z_{st}$ is a $\left(S+R_{0}+R_{1}-1\right)$ -dimensional vector with a 1 in the t th position and zeros elsewhere, and $\beta =(\beta_{1},\ldots ,\beta_{S+R_{0}+R_{1}-1}{)}^{T}$ so that $Z_{st}\beta =\beta_{t}$ . A linear time effect is returned if $Z_{st}=(1,t)$ and $\beta =(\beta_{1},\beta_{2}{)}^{T}$ so that $Z_{st}\beta =\beta_{1}+t\beta_{2}$ .

#### 2.2.3 Intracluster correlation structures

Several forms for $V_{CP}$ have been proposed for longitudinal cluster randomised trials, differing in the extent to which the off-diagonal terms, i.e., the covariance between outcomes from subjects in the same cluster measured in different periods, vary over the periods. A general form is induced by the following relationships: $\text{var}({\text{CP}}_{\text{skt}})={\sigma_{C}}^{2}$ and $\text{cov}({\text{CP}}_{skt},{\text{CP}}_{skt^{\prime }})=r_{tt^{\prime }}{\sigma_{C}}^{2}$ , for $0<r_{tt^{\prime }}\leq 1$ , so that the diagonal elements of $V_{CP}$ are given by ${\sigma_{C}}^{2}$ and the off-diagonal elements are given by $r_{tt^{\prime }}{\sigma_{C}}^{2}$ . These variance components, together with the other variance components in model (1), yield the intracluster correlation parameters. The within-period intracluster correlation (or within-period ICC) describes the correlation between the outcomes of subjects measured in the same cluster in the same period and is given by $\text{corr}(Y_{skti},Y_{skti^{\prime }})={\sigma_{C}}^{2}/({\sigma_{C}}^{2}+{\sigma_{\epsilon }}^{2}):=\rho_{0}$ . The between-period intracluster correlation describes the correlation between different subjects’ outcomes in the same cluster measured in different periods of the trial, given by $\text{corr}(Y_{skti},Y_{skt^{\prime }i^{\prime }})=(r_{tt^{\prime }}{\sigma_{C}}^{2})/({\sigma_{C}}^{2}+{\sigma_{\epsilon }}^{2})=r_{tt^{\prime }}\rho_{0}$ .

The block-exchangeable, or constant between-period intracluster correlation structure, is obtained if we assume that $r_{tt^{\prime }}=r$ , 0<r≤1 for all pairs $t\neq t^{\prime }$ . This model was introduced by Hooper et al.^15^ and Girling and Hemming,^16^ and has been considered in many analyses of stepped wedge and related trials. This model assumes that the correlations between all pairs of subjects’ outcomes in different periods are the same and do not depend on the length of time between their measurements. An alternative correlation structure allows for these correlations to decrease as the time between subjects’ periods of measurement increases. This decreasing correlation over the trial's periods is encoded by the discrete-time decay correlation structure and is returned if we assume $r_{tt^{\prime }}=r^{\left|t-t^{\prime }\right|}$ , 0<r≤1 , where *r* is called the cluster autocorrelation.^17^ This structure has been primarily discussed in the context of stepped wedge designs but is also applicable to other longitudinal cluster randomised trial designs. A special case of these intracluster correlation structures is returned when r=1 , yielding an exchangeable correlation structure.^1^

#### 2.2.4. Cluster-period mean level model

For both time parameterisations and the intracluster correlation structures described above, time is synonymous with study period, and is treated as a discrete phenomenon. As such, model (1) can be collapsed to cluster-period means without loss of information.^18^ Letting ${\bar{Y}}_{skt}=\frac{1}{m}\sum_{i=1}^{m}Y_{skti}$ denote the mean of all observations in sequence *s* in cluster *k* during period *t*, model (1) collapses to:

(2) $$\begin{array}{r} {\bar{Y}}_{skt}=Z_{st}\beta +X_{st}\theta +CP_{skt}+\epsilon_{skt},\epsilon_{skt}∼N\left(0,\frac{{\sigma_{\epsilon }}^{2}}{m}\right),CP_{sk}∼N_{R_{0}+R_{1}}(0,V_{CP}) \end{array}$$

where $\epsilon_{skt}=\frac{1}{m}\sum_{i=1}^{m}\epsilon_{skti}$ . We denote the $\left(R_{0}+R_{1}\right)$ -dimensional vector of cluster-period means for cluster *k* assigned to sequence *s* as ${\bar{Y}}_{sk}=({\bar{Y}}_{sks},\ldots ,{\bar{Y}}_{sk,s+R_{0}+R_{1}-1}{)}^{T}$ . The $\left(R_{0}+R_{1})\times (R_{0}+R_{1}\right)$ covariance matrix associated with this vector is then given by:

$$\begin{array}{r} \text{cov}({\bar{Y}}_{sk})=V_{*}=V_{CP}+\frac{{\sigma_{\epsilon }}^{2}}{m}I_{R_{0}+R_{1}} \end{array}$$

where $I_{a}$ is the a×a identity matrix.

#### 2.2.5. Modifications for cohort designs

While the models above are appropriate for a sampling scheme where different subjects are measured in each period and just one measurement is taken on each subject (e.g., a ‘repeated cross sectional’ sampling scheme),^19^ a cohort design could be modelled by including a subject-level random effect term to account for repeated measures on the same subject. Specifically, we could include the term $u_{ski}$ in model (1), where $u_{ski}∼N(0,\sigma_{u}^{2})$ , which would become $u_{sk}=\frac{1}{m}\sum_{i=1}^{m}u_{ski}$ in model (2), where $u_{sk}∼N\left(0,\frac{\sigma_{u}^{2}}{m}\right)$ . The inclusion of this term would imply exchangeability between a subject's repeated measures, conditional on cluster and cluster-period, although more sophisticated relationships such as decaying correlation encoded by an autoregressive structure could be assumed. This additional random effect would then yield different correlation parameters, as $\sigma_{u}^{2}$ would appear in the denominator of $\rho_{0}$ , and we would have an additional correlation parameter, representing the correlation between observations on the same subject at different periods, e.g., $\rho_{u}\;:=\;\mathrm{corr}(Y_{skti},Y_{skt^{\prime }i})={\sigma_{u}}^{2}/({\sigma_{C}}^{2}+{\sigma_{u}}^{2}+{\sigma_{\epsilon }}^{2})$ . The expressions we derive below for the variance of the treatment effect estimator are applicable to cohort designs through the appropriate specification of the covariance matrix of the cluster-period means, $\text{cov}({\bar{Y}}_{sk})=V_{*}$ .

### 2.3. Variance of the treatment effect estimator for general staircase designs

We present a formula for the variance of the treatment effect estimator for general staircase designs, $\text{var}(\hat{\theta }{)}_{SC}$ , when $\hat{\theta }$ is the generalised least squares estimator. This variance is a key ingredient in sample size and power calculations.^18^ Let $X_{s}=(X_{ss},\ldots ,X_{s,s+R_{0}+R_{1}-1}{)}^{T}$ denote the vector of treatment indicators for the periods of measurement in sequence *s*. Since for the designs we consider in this paper the vector of treatment indicators is common across sequences, we let $X_{s}=X$ . Let $Z_{s}=(Z_{ss},\ldots ,Z_{s,s+R_{0}+R_{1}-1}{)}^{T}$ denote the design matrix for sequence *s* encoding the parameterisation of the time effects and $V_{*}$ denote the $\left(R_{0}+R_{1})\times (R_{0}+R_{1}\right)$ covariance matrix for a cluster over its periods of measurement.

We show in Section A of the Supporting Information that the variance of the treatment effect estimator for a general staircase design can be represented as:

(3) $$\begin{array}{r} \text{var}(\hat{\theta }{)}_{SC}=\frac{1}{\text{SK}}{\left[X^{T}{V_{*}}^{-1}X-\frac{1}{S}X^{T}{V_{*}}^{-1}\sum_{s=1}^{S}Z_{s}{\left(\sum_{s=1}^{S}{Z_{s}}^{T}{V_{*}}^{-1}Z_{s}\right)}^{-1}\sum_{s=1}^{S}{Z_{s}}^{T}{V_{*}}^{-1}X\right]}^{-1}. \end{array}$$

Further simplification of this expression is limited by the ability to reason about the elements of the matrix obtained by inverting the inner term, $\sum_{s=1}^{S}{Z_{s}}^{T}{V_{*}}^{-1}Z_{s}$ , for an arbitrary number of sequences *S* and complex forms of the covariance matrix $V_{*}$ . We return to this point in Section 5. However, the basic staircase with $R_{0}=R_{1}=1$ permits explicit expressions which we present in the next section.

## 3. Basic staircase designs: An in-depth look

### 3.1. Variance and correlation parameters for the basic staircase

For the basic staircase design, SC(S,K,1,1) , the vector of treatment indicators is given by $X=(0,1{)}^{T}$ and $V_{*}$ is a 2×2 covariance matrix. Suppose $V_{*}$ contains elements *a* on the diagonal, representing the variance of a cluster-period mean, and *b* on the off-diagonal, representing the covariance between the cluster-period means. For model (2), these elements would be $a={\sigma_{C}}^{2}+\sigma_{\epsilon }^{2}/m$ and $b=r{\sigma_{C}}^{2}$ for either the block-exchangeable or discrete-time decay intracluster correlation structure; these structures are identical when there are only two periods of measurement per sequence as in a basic staircase design. We could also represent these elements in terms of the correlation parameters, $\rho_{0}$ and r , and cluster-period size, *m*. Assuming the total variance ${\sigma_{C}}^{2}+{\sigma_{\epsilon }}^{2}=1$ , we would have $a=\frac{1+(m-1)\rho_{0}}{m}$ and $b=r\rho_{0}$ . We can then write the correlation between cluster-period means, $\text{corr}({\bar{Y}}_{sks},{\bar{Y}}_{sk,s+1})$ as $\psi =\frac{b}{a}=\frac{mr\rho_{0}}{1+(m-1)\rho_{0}}$ . Although the variance of the treatment effect estimator for the general staircase design does not have a tractable form, we are able to reason about the treatment effect estimator and obtain explicit expressions for the variance of the treatment effect estimator for the basic staircase. We first assume categorical period effects and then assume a linear effect of time over the trial periods.

### 3.2. Variance of the treatment effect estimator, four-sequence design

#### 3.2.1. Treatment effect estimator

**Figure 2.**
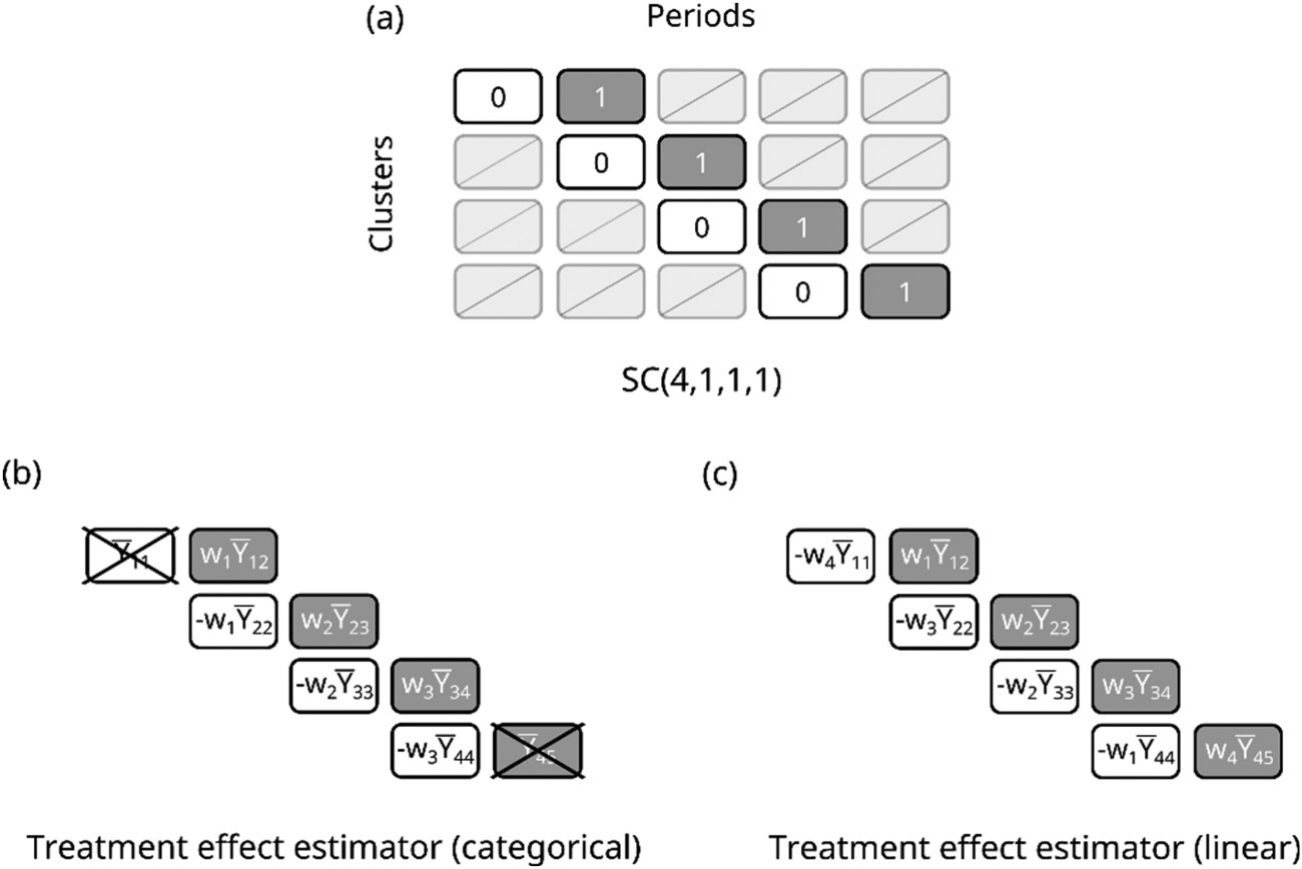
(a) Design schematic for a four-sequence basic staircase design; (b) visualisation of the treatment effect estimator in terms of the mean outcomes from the measured cluster-periods, assuming categorical period effects; (c) visualisation of the treatment effect estimator in terms of the mean outcomes from the measured cluster-periods, assuming a linear time effect.

First consider the SC(4,1,1,1) design, a four-sequence basic staircase design with one cluster per sequence (Figure 2(a)). Let ${\bar{Y}}_{st}$ denote the mean outcome corresponding to sequence *s* in period *t*. We can represent the estimator for θ as a linear combination of the means ${\bar{Y}}_{st}$ for all measured cluster-periods:

$$\begin{array}{r} \hat{\theta }=w_{11}{\bar{Y}}_{11}+w_{12}{\bar{Y}}_{12}+w_{22}{\bar{Y}}_{22}+w_{23}{\bar{Y}}_{23}+w_{33}{\bar{Y}}_{33}+w_{34}{\bar{Y}}_{34}+w_{44}{\bar{Y}}_{44}+w_{45}{\bar{Y}}_{45}. \end{array}$$

In the subsections to follow, we derive explicit expressions for the best linear unbiased treatment effect estimator and its variance, first assuming categorical period effects and then assuming a linear effect of time.

#### 3.2.2. Categorical period effects

Under model (2) and assuming categorical period effects such that $Z_{st}\beta =\beta_{t}$ , $\hat{\theta }$ has expectation

$$\begin{array}{r} E(\hat{\theta })=(w_{12}+w_{23}+w_{34}+w_{45})\theta +w_{11}\beta_{1}+(w_{12}+w_{22})\beta_{2}+(w_{23}+w_{33})\beta_{3}+(w_{34}+w_{44})\beta_{4}+w_{45}\beta_{5}. \end{array}$$

Since we want $\hat{\theta }$ to be an unbiased estimator of θ , the following conditions must hold:

(i)
$w_{11}=0$ and $w_{45}=0$
(ii)
$w_{12}+w_{22}=w_{23}+w_{33}=w_{34}+w_{44}=0$

(iii)
$w_{12}+w_{23}+w_{34}+w_{45}=1$ .

Condition (i) implies that the outcomes from the cluster-periods in the first and last periods of the design, ${\bar{Y}}_{11}$ and ${\bar{Y}}_{45}$ , are not used at all to estimate the treatment effect. This holds for larger basic staircase designs: When a categorical period effect is included in the model, the outcomes from the cells in the first and last periods of the design do not contribute to the estimation of the treatment effect.

Condition (ii) suggests that the weights on cells in the same period but different clusters have the same magnitude but opposite signs. We can then rewrite the general treatment effect estimator in terms of three unique weights, letting $w_{1}=w_{12}=-w_{22}$ , $w_{2}=w_{23}=-w_{33}$ , and $w_{3}=w_{34}=-w_{44}$ :

$$\begin{array}{r} \hat{\theta }=w_{1}({\bar{Y}}_{12}-{\bar{Y}}_{22})+w_{2}({\bar{Y}}_{23}-{\bar{Y}}_{33})+w_{3}({\bar{Y}}_{34}-{\bar{Y}}_{44}) \end{array}$$

where $w_{1}+w_{2}+w_{3}=1$ . Figure 2(b) depicts this expression in the context of the design schematic.

Then the variance of the treatment effect estimator is given by

$$\begin{array}{rl} \text{var}(\hat{\theta }) & =w_{1}^{2}\left[\text{var}({\bar{Y}}_{12})+\text{var}({\bar{Y}}_{22})\right]+w_{2}^{2}\left[\text{var}({\bar{Y}}_{23})+\text{var}({\bar{Y}}_{33})\right]\\ & \;+w_{3}^{2}\left[\text{var}({\bar{Y}}_{34})+\text{var}({\bar{Y}}_{44})\right]-2w_{1}w_{2}\text{cov}({\bar{Y}}_{22},{\bar{Y}}_{23})-2w_{2}w_{3}\text{cov}({\bar{Y}}_{33},{\bar{Y}}_{34})\\ & =2a(w_{1}^{2}+w_{2}^{2}+w_{3}^{2})-2b(w_{1}w_{2}+w_{2}w_{3})\\ & =2a\left[w_{1}^{2}+w_{2}^{2}+w_{3}^{2}-\psi (w_{1}w_{2}+w_{2}w_{3})\right] \end{array}$$

where *a* represents the common variance of a cluster-period mean and ψ represents the correlation between the two cluster-period means within a cluster.

We can then find the weights that give lowest variance by minimising the expression $a[w_{1}^{2}+w_{2}^{2}+w_{3}^{2}-\psi (w_{1}w_{2}+w_{2}w_{3})]$ using Lagrange multiplier equations, with the constraint from above that $w_{1}+w_{2}+w_{3}-1=0$ . This approach (detailed in Supporting Information Section B.1) yields the following weights:

$$\begin{array}{r} w_{1}=w_{3}=\frac{1+\frac{\psi }{2}}{3+2\psi },w_{2}=\frac{1+\psi }{3+2\psi }. \end{array}$$

Table 1 displays these weights in terms of the correlation ψ between cluster-period means, and for three different scenarios: when the cluster-period means are uncorrelated (ψ=0) , moderately correlated (ψ=1/2) , and fully correlated (ψ=1) . If the outcomes from the cluster-periods within a cluster are uncorrelated, then all but the outermost cluster-periods are weighted with the same magnitude to estimate the treatment effect. As the correlation between cluster-period means increases, the magnitude of the weights on the innermost cluster-periods increases slightly, as these cluster-periods are correlated in both directions with cluster-periods in the adjacent periods.

**Table 1.** Weights on cluster-period mean differences, depending on the correlation ψ between cluster-period means.

Weight	Cluster-period mean difference	General	ψ=0	ψ=1/2	ψ=1
$w_{1}$	${\bar{Y}}_{12}-{\bar{Y}}_{22}$	$\frac{1+\frac{\psi }{2}}{3+2\psi }$	$\frac{1}{3}$	$\frac{5}{16}$	$\frac{3}{10}$
$w_{2}$	${\bar{Y}}_{23}-{\bar{Y}}_{33}$	$\frac{1+\psi }{3+2\psi }$	$\frac{1}{3}$	$\frac{6}{16}$	$\frac{4}{10}$
$w_{3}$	${\bar{Y}}_{34}-{\bar{Y}}_{44}$	$\frac{1+\frac{\psi }{2}}{3+2\psi }$	$\frac{1}{3}$	$\frac{5}{16}$	$\frac{3}{10}$

Finally, plugging these weights back into the previous variance expression and simplifying gives

(4) $$\begin{array}{r} \text{var}(\hat{\theta }{)}_{SC(4,1,1,1),\;cat}=\frac{a(2-\psi^{2})}{3+2\psi }. \end{array}$$

Note that if *K* clusters were randomised to each of the *S* sequences of the design, then the variance of the treatment effect estimator would simply be reduced by a factor of 1/K .

#### 3.2.3. Linear period effects

Using a similar approach to that outlined in Section 3.2.2, we show in Supporting Information Section B.2 that when assuming linear rather than categorical period effects, for a four-sequence basic staircase, the treatment effect estimator can be written as a linear combination of differences between centrosymmetric pairs of cluster-period cells:

$$\begin{array}{r} \hat{\theta }=w_{1}({\bar{Y}}_{12}-{\bar{Y}}_{44})+w_{2}({\bar{Y}}_{23}-{\bar{Y}}_{33})+w_{3}({\bar{Y}}_{34}-{\bar{Y}}_{22})+w_{4}({\bar{Y}}_{45}-{\bar{Y}}_{11}) \end{array}$$

where the weights have the following values:

$$\begin{array}{r} w_{1}=\frac{4}{10},w_{2}=\frac{3}{10},\;w_{3}=\frac{2}{10},\;w_{4}=\frac{1}{10}. \end{array}$$

The treatment effect estimator can then be written as

$$\begin{array}{r} \hat{\theta }=\frac{1}{10}\left[4({\bar{Y}}_{12}-{\bar{Y}}_{44})+3({\bar{Y}}_{23}-{\bar{Y}}_{33})+2({\bar{Y}}_{34}-{\bar{Y}}_{22})+({\bar{Y}}_{45}-{\bar{Y}}_{11})\right]. \end{array}$$

Note that unlike when categorical period effects are assumed, all cluster-period means contribute to the treatment effect estimate when linear period effects are assumed. Moreover, the weights on the cluster-period means do not depend on the correlation between cluster-period means, ψ . A depiction of the treatment effect estimator in the context of the design schematic is shown in Figure 2(c).

Finally, the variance of the treatment effect estimator is given by

(5) $$\begin{array}{r} \text{var}(\hat{\theta }{)}_{SC(4,1,1,1),\;lin}=\frac{a(3-2\psi )}{5}. \end{array}$$

As before, if *K* clusters were randomised to each of the *S* sequences of the design, then the variance of the treatment effect estimator would be reduced by a factor of 1/K .

### 3.3. Variance of the treatment effect estimator, S -sequence design

#### 3.3.1. Obtaining general results

We consider two different approaches to obtaining explicit expressions for the variance of the treatment effect estimator for a basic staircase design with *S* sequences: extending the approach from Section 3.2 or by simplifying expression (3) using matrix algebra and results on explicit inverses of particular matrices. We briefly describe these approaches in the subsections to follow, with more detail available in Supporting Information Section C.

#### 3.3.2. Categorical period effects

Using a similar approach to that used in Section 3.2.2 for a four-sequence design, we show in Section C.1 of the Supporting Information that for a general S -sequence basic staircase design where categorical period effects are assumed, the outcomes from the cluster-periods in the first and last periods have weights of zero and therefore do not contribute to the estimation of the treatment effect. In addition, the set of weights within each period sum to zero. Therefore, the treatment effect estimator can be represented as the weighted sum of several ‘vertical comparisons’ between the cluster-period means within each of the intermediate periods of the trial:

$$\hat{\theta }=\sum_{s=1}^{S-1}w_{s}({\bar{Y}}_{s,s+1}-{\bar{Y}}_{s+1,s+1}).$$

Extending the approach from Section 3.2.2, we show in Section C.1 of the Supporting Information that the resulting variance of the treatment effect estimator can be represented as

**Figure 3.**
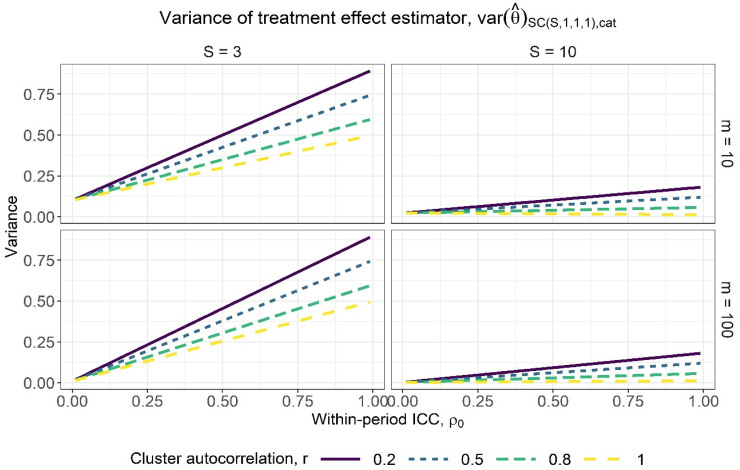
Variance of the treatment effect estimator for varying within-period intracluster correlation (ICC) values, assuming categorical period effects, for a basic staircase design with three and 10 sequences (columns) and cluster-period sizes of 10 and 100 (rows), where each subject is measured just once. The lines within each subplot correspond to different cluster autocorrelation values.


(6) $$\text{var}(\hat{\theta }{)}_{SC(S,K,1,1),\;cat}=\frac{2a{\left(1-\psi \right)}^{2}}{K\left[S(1-\psi )-\sqrt{1-\psi^{2}}\frac{{\left(1+\sqrt{1-\psi^{2}}\right)}^{S}-\psi^{S}}{{\left(1+\sqrt{1-\psi^{2}}\right)}^{S}+\psi^{S}}\right]}$$

where, as described in Section 3.1, $a=\frac{1+(m-1)\rho_{0}}{m}$ and $\psi =\frac{mr\rho_{0}}{1+(m-1)\rho_{0}}$ .

Another approach for deriving the variance is by simplifying expression (3) with results appropriate for the basic staircase design. Assuming categorical period effects, the matrices encoding the time effects, $Z_{s}$ , s=1,…,S , are 2×(S+1) -dimensional matrices comprised entirely of zeros except for a 2×2 identity matrix starting in column *s*. The innermost term in expression (3), $\sum_{s=1}^{S}{Z_{s}}^{T}{V_{*}}^{-1}Z_{s}$ , is a tridiagonal matrix comprised of $\frac{-b}{a^{2}-b^{2}}$ on the super- and sub-diagonals, with elements $\frac{a}{a^{2}-b^{2}}$ in the first and last positions of the diagonal and $\frac{2a}{a^{2}-b^{2}}$ in the inner positions of the diagonal. Utilising results in Tan^20^ for inverting real symmetric tridiagonal matrices of this form, we show in Section C.2 of the Supporting Information that the resulting variance of the treatment effect estimator is also given by expression (6).

**Figure 4.**
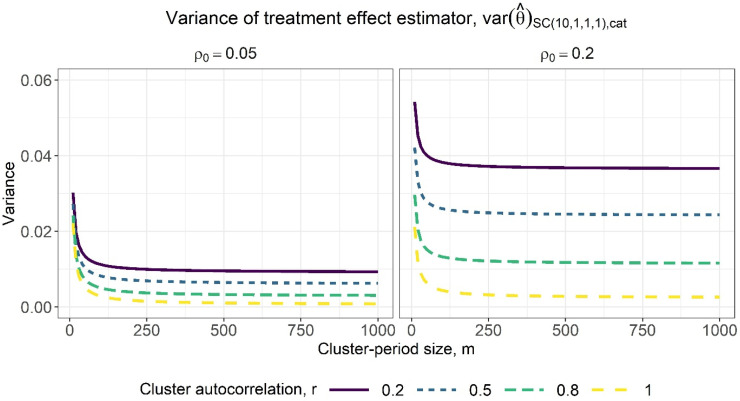
Variance of the treatment effect estimator for varying cluster-period sizes, assuming categorical period effects, for a basic staircase design with 10 sequences, where each subject is measured just once, and for within-period ICC values of 0.05 (left) and 0.2 (right). The lines within each subplot correspond to different cluster autocorrelation values.

Figure 3 displays the variance of the treatment effect estimator against the full range of within-period ICC values under the assumption of categorical period effects, for basic staircase designs with differing numbers of sequences (columns) and cluster-period sizes (rows), for several different cluster autocorrelation values. There appears to be a near-linear relationship between the variance of the treatment effect estimator and the within-period ICC. It tends to be an increasing relationship, however we see a slight decreasing relationship for the design with 10 sequences, a cluster-period size of 10, and for a cluster autocorrelation of r=1 as for exchangeable intracluster correlation. The variance of the treatment effect estimator is lower for higher autocorrelation values: less decay in correlation from one period to the next means that subjects’ outcomes from the control period will generally be more similar to those in the intervention period, making it easier to attribute any difference to the treatment effect. Variances are lower for designs with more sequences, as these designs have more clusters and therefore more measurements with which to estimate the treatment effect. Increasing the cluster-period size yields a slight decrease in the variance, although there is less benefit to measuring more subjects in a cluster-period as the within-period ICC increases: the subjects are more similar and so each additional subject offers less new information about the treatment effect. Furthermore, Figure 4 illustrates that there are rapidly diminishing returns to increasing the cluster-period size. After an initial sharp decrease in the variance of the treatment effect estimator as the cluster-period size increases, the rate of decrease of the variance then quickly slows such that little precision would be gained from designs with increasingly large cluster-period sizes.

#### 3.3.3. Linear period effects

In a generalisation of the results in Section 3.2.3 for a four-sequence design, the treatment effect estimator for an *S*-sequence basic staircase design where linear period effects are assumed can similarly be written as a weighted sum of the differences between centrosymmetric cluster-period cells, i.e., ${\bar{Y}}_{st}$ and ${\bar{Y}}_{S+1-s,T+1-t}$ :

$$\begin{array}{r} \hat{\theta }=\sum_{s=1}^{S}w_{s}({\bar{Y}}_{s,s+1}-{\bar{Y}}_{S+1-s,S+1-s}). \end{array}$$

To obtain an explicit expression for the variance of the treatment effect estimator, we simplify expression (3) using results appropriate for an *S*-sequence basic staircase design where a linear time effect over the trial periods is assumed. The matrices encoding the time effects, $Z_{s}$ , s=1,…,S , are 2×2 -dimensional matrices comprised of ones in the first column and the elements *s* and s+1 in the second column. The inner term in expression (3), $\sum_{s=1}^{S}{Z_{s}}^{T}{V_{*}}^{-1}Z_{s}$ , remains a 2×2 matrix and hence is straightforward to invert. We show in Section C.3 of the Supporting Information that the resulting variance of the treatment effect estimator is given by:

(7) $$\begin{array}{r} \text{var}(\hat{\theta }{)}_{SC(S,K,1,1),\;lin}=\frac{2a\left[(S^{2}+2)-(S^{2}-4)\psi \right]}{KS(S^{2}-1)}. \end{array}$$

Since $a=\frac{1+(m-1)\rho_{0}}{m}$ and $\psi =\frac{mr\rho_{0}}{1+(m-1)\rho_{0}}$ , this variance expression can also be written as a function of the intracluster correlation parameters $\rho_{0}$ and *r* and cluster-period size m :

(8) $$\begin{array}{r} \text{var}(\hat{\theta }{)}_{SC(S,K,1,1),\;lin}=\frac{2}{KS(S^{2}-1)}\left\{\left[\left(1-\frac{1}{m}\right)(S^{2}+2)-(S^{2}-4)r\right]\rho_{0}+\frac{1}{m}(S^{2}+2)\right\}. \end{array}$$

**Figure 5.**
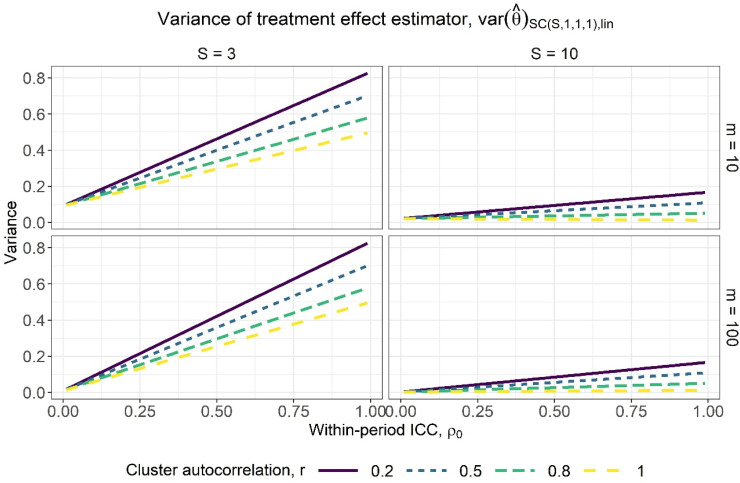
Variance of the treatment effect estimator for varying within-period intracluster correlation (ICC) values, assuming linear period effects, for a basic staircase design with three and 10 sequences (columns) and cluster-period sizes of 10 and 100 (rows), where each subject is measured just once. The lines within each subplot correspond to different cluster autocorrelation values.

While the variance is linear in $\rho_{0}$ , the coefficient on $\rho_{0}$ may be positive or negative, depending on the trial configuration: greater similarity between participants’ outcomes within a cluster may increase or decrease the precision of the treatment effect. This coefficient would be negative in settings where $r>\left(1-\frac{1}{m}\right)\frac{S^{2}+2}{S^{2}-4}$ . One such example is a basic staircase design with S=10 sequences, a cluster-period size of m=10 , and a cluster autocorrelation of r>0.96 which includes exchangeable intracluster correlation ( r=1 , shown in Figure 5). Under an assumption of exchangeable intracluster correlation, these settings could be denoted by a design with number of sequences $S>\sqrt{2(3m-1)}$ , e.g., eight or more sequences for a cluster-period size of 10, and 25 or more sequences for a cluster-period size of 100.

**Figure 6.**
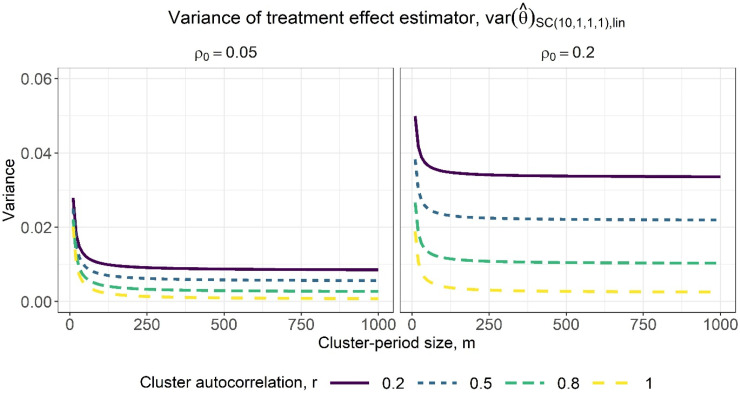
Variance of the treatment effect estimator for varying cluster-period sizes, assuming linear period effects, for a basic staircase design with 10 sequences, where each subject is measured just once, and for within-period intracluster correlation (ICC) values of 0.05 (left) and 0.2 (right). The lines within each subplot correspond to different cluster autocorrelation values.

Figure 5 displays the variance of the treatment effect estimator from expression (8) against a range of within-period ICC values, for basic staircase designs with differing numbers of sequences (columns) and cluster-period sizes (rows), for several different cluster autocorrelation values. The observations above for categorical period effects are borne out: higher values of *r* and lower values of $\rho_{0}$ correspond to higher precision. There are again diminishing returns to increasing the cluster-period size, with the benefit quickly tapering off (Figure 6). Note that by a close inspection of Figures 3 and 5, for the same combination of within-period ICC and cluster autocorrelation values, the variance of the treatment effect estimator under the assumption of linear period effects (Figure 5) is slightly lower than under the assumption of categorical period effects (Figure 3). This is to be expected given the assumption of linear period effects requires the estimation of fewer parameters than the assumption of categorical period effects.

## 4. Sample size and power calculations

### 4.1. Sample size and power calculations using formulae

The variance expressions provided in Sections 2.3 and 3.3, together with a standard power formula, can be used to calculate the power of a trial for a desired effect size or the required number of clusters for a particular cluster-period size and desired level of power. Expression (3) is applicable to general staircase designs, and expressions (6) and (7) are applicable to basic staircase designs, with terms *a* and ψ again representing the variance of and correlation between cluster-period means, respectively. A standard power formula for cluster randomised trials^8^ is given by

$$\begin{array}{r} z_{\beta }=\frac{\left|\delta \right|}{\sqrt{\text{var}(\hat{\theta })}}-z_{\alpha /2} \end{array}$$

so that

$$\begin{array}{r} \text{Power}=1-\beta =\Phi \left(\frac{\left|\delta \right|}{\sqrt{\text{var}(\hat{\theta })}}-z_{\alpha /2}\right) \end{array}$$

where Φ is the cumulative standard Normal distribution, δ is the target effect size, α is the two-sided significance level of interest, $z_{\gamma }$ is the value for the standard Normal distribution corresponding to right tail area γ , and $\mathrm{var}(\hat{\theta })$ is the variance of the treatment effect estimator for the trial design of interest.

### 4.2. Staircase trial example

**Figure 7.**
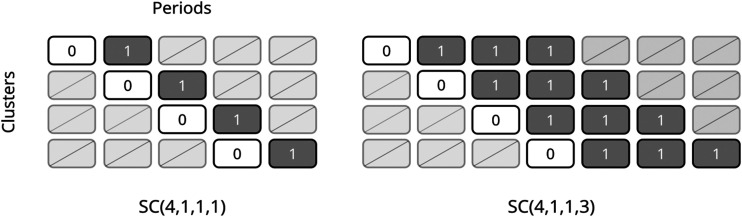
Design schematics for a basic staircase design with one cluster assigned to each of four unique sequences (left) and an imbalanced staircase design with one control period followed by three intervention periods in each sequence and one cluster assigned to each of four unique sequences (right).

One example of a planned staircase design is described by White-Traut et al.^21^ as a cluster randomised trial across four neonatal intensive care units (NICUs) to test whether a behavioural intervention for preterm infants leads to improved growth while in the NICU. The researchers specified a design with four unique sequences and four measurement periods in each sequence, with each sequence consisting of one control period followed by three intervention periods and each sequence commencing data collection in a different period. Labelled an ‘incomplete stepped wedge’ in the protocol paper, using the terminology and notation in this paper, this design would be deemed an ‘imbalanced staircase’, described in our notation as SC(4,1,1,3) . While we will not replicate their exact study design which appears to involve multiple cohorts, we note that for their power calculations, the authors assumed a standardised effect size of 0.5, two-sided significance level of 0.05, and an ICC of 0.1. The authors anticipated that between 62 and 252 infants would be eligible for the trial in a 6-month period in the participating NICU clusters. Generally, we would recommend that staircase designs, or any cluster randomised trial design, be conducted with a larger number of clusters than the four in the White-Traut et al. trial, as the asymptotic normality of the treatment effect estimator that we have considered relies on a large number of clusters. However, here we apply our derived expressions to two designs with four clusters, as inspired by this trial (Figure 7).

We first consider a basic staircase design for this trial setting and aim: SC(4,1,1,1) . We will suppose that 50 subjects per cluster per period could feasibly be included in the trial, with each subject measured just once. Then assuming an exchangeable intracluster correlation structure (r=1) , we can calculate the power of a basic staircase design with S=4 , K=1 , $\rho_{0}=0.1$ and m=50 using equations (4) or (5) to first obtain the variance of the treatment effect estimator and then use a standard expression for power. The variance *a* of a cluster-period mean and correlation ψ between cluster-period means are given by $a=\frac{1+(m-1)\rho_{0}}{m}=0.118$ and $\psi =\frac{mr\rho_{0}}{1+(m-1)\rho_{0}}=0.847$ .

If categorical period effects were assumed, the variance of the treatment effect estimator for this design obtained from (4) is

$$\begin{array}{r} \text{var}(\hat{\theta }{)}_{SC(4,1,1,1),\;cat}=\frac{\left(0.118)(2-0.847^{2}\right)}{3+2(0.847)}=0.0322, \end{array}$$

corresponding to 79.6% power to detect an effect size of 0.5:

$$\begin{array}{r} \Phi \left(\frac{\left|-0.5\right|}{\sqrt{0.0322}}-1.96\right)=0.796. \end{array}$$

Assuming linear period effects instead, the variance of the treatment effect estimator for this design obtained from (5) is

$$\begin{array}{r} \text{var}(\hat{\theta }{)}_{SC(4,1,1,1),\;lin}=\frac{\left(0.118)(3-2(0.847)\right)}{5}=0.0308, \end{array}$$

corresponding to 81.3% power:

$$\begin{array}{r} \Phi \left(\frac{\left|-0.5\right|}{\sqrt{0.0308}}-1.96\right)=0.813. \end{array}$$

Note that equations (6) and (7) could instead be used to calculate the variance of the treatment effect estimator for this design, and for basic staircase designs with other numbers of sequences.

If a staircase design with more than one control and/or intervention period were of interest, such as a SC(4,1,1,3) design with one control period followed by three intervention periods in each of four sequences, then equation (3) can instead be used to calculate the variance of the treatment effect estimator:

$$\begin{array}{r} \text{var}(\hat{\theta }{)}_{SC(4,1,1,3)}=\frac{1}{4}{\left[X^{T}{V_{*}}^{-1}X-\frac{1}{4}X^{T}{V_{*}}^{-1}\sum_{s=1}^{4}Z_{s}{\left(\sum_{s=1}^{4}{Z_{s}}^{T}{V_{*}}^{-1}Z_{s}\right)}^{-1}\sum_{s=1}^{4}{Z_{s}}^{T}{V_{*}}^{-1}X\right]}^{-1} \end{array}$$

where $X=(0,1,1,1{)}^{T}$ , $V_{*}$ is a 4×4 matrix consisting of elements a=0.118 along the diagonal and b=0.1 in each off-diagonal cell, and $Z_{s}$ is either a 4×7 matrix containing a 4×4 identity matrix starting in column *s* and zeros elsewhere if categorical period effects are assumed, or a 4×2 matrix made up of ones in the first column and integers *s* up to s+3 in the second column if linear period effects are instead assumed. The variance of the treatment effect estimator assuming categorical period effects is 0.017 corresponding to 96.9% power to detect an effect size of 0.5, and the variance assuming linear period effects is 0.014 corresponding to 98.8% power.

### 4.3. Available tools for calculating required sample size and power

While statisticians and researchers could use our results to manually calculate sample size and power for their studies, some may wish to use existing tools to do so. Some, but not all, of the existing tools and software appropriate for stepped wedge and other cluster randomised trial designs, can also be used to calculate required sample size and power for staircase designs.^22^ A requirement is that the tool or software can accommodate incomplete designs with periods in which no measurements are taken in some clusters, either by allowing users to specify an incomplete design schematic directly or by specifying an ‘inclusion’ matrix to exclude certain cluster-period cells from a standard design schematic. Further requirements to cover the scenarios in this paper are options for different intracluster correlation structures including the block-exchangeable and discrete-time decay correlation structures, and both categorical and linear forms for time. As of the time of writing, tools that fulfil all of the above requirements include the SteppedPower R package^23^ and a SAS macro called %CRTFASTGEEPWR^24^; a freely available web app called the Shiny CRT calculator^25^ fulfils all of the above except allowing different forms for time.^22^

The main power function in the SteppedPower R package^23^ has an ‘incomplete’ argument: a scalar input retains the specified number of pre- and post-switch periods in each treatment sequence from a stepped wedge design, or a matrix input can be used to denote inclusion (cells with a value of 1) or exclusion (cells with a value of 0) of the cluster-period cells from a complete stepped wedge design schematic. A balanced staircase design, for example, could be specified by defining a complete stepped wedge along with ‘incomplete = 2’; however, the first and last sequences of the resulting design are truncated, lacking an initial control period and final intervention period, respectively. We note that this will not affect the variance of the treatment effect estimator or power when assuming categorical period effects but it will affect the resulting calculation if assuming linear period effects. We expand upon this point in Section 5. The %CRTFASTGEEPWR SAS macro^24^ takes a matrix for the design specification, allowing incomplete designs through the use of a ‘2’ for cluster-period cells in which no measurements are taken. The Shiny CRT calculator web app^25^ allows users to upload a design schematic as a CSV file. Staircase designs can be specified similarly to the design schematics in Figure 1, with empty cells for cluster-periods in which no measurements are taken. This app does not allow users to specify different time parameterisations, but rather assumes categorical period effects for all calculations. Sample code for each of these implementations is provided in Appendix A.

## 5. Discussion

In this article, we formally introduced the staircase design, considering the properties of this design when a linear model for the outcome is assumed, focusing on the properties of the basic staircase design with one pre- and one post-switch measurement period. We derived a simplified analytical expression for the variance of the treatment effect estimator for a general staircase design to enable appropriate sample size and power calculations for these designs. In addition, we derived explicit expressions for the variance of the treatment effect estimator for the basic staircase design under assumptions of categorical and linear period effects.

A staircase design is a pragmatic alternative to a stepped wedge design. Since sequences in a staircase design contain only a limited number of pre- and post-switch measurement periods, this design can potentially be much less burdensome and expensive than a stepped wedge. These reduced data collection requirements would likely be more enticing for participating clusters and could carry a reduced risk of attrition of clusters during the trial. Beyond the appeal of the limited number of measurement periods, the staggering of these sequences means that each cluster would receive the intervention sooner upon commencing data collection. This could also be appealing for participating clusters, but in practice it would mean that not all clusters would be actively involved at all times. It is possible that this reduced engagement could cause inactive clusters to lose interest and withdraw from the trial.

The implications of clustering for the efficiency of a basic staircase design is similar to other longitudinal cluster randomised trial designs in some ways but different in others. The variance of the treatment effect estimator is a linear or near-linear function of the within-period ICC. Under a discrete-time decay correlation structure, the stepped wedge design has a nonlinear relationship between the variance and within-period ICC.^17^ As with other cluster randomised trial designs, a basic staircase design sees diminishing returns from increasing the cluster-period size: beyond a certain cluster-period size, minimal useful information about the treatment effect can be gained by measuring more subjects in a cluster-period.^26^

Unlike the stepped wedge design for which assuming categorical or linear time effects yields the same variance expression and hence same calculated sample size for the same parameter value inputs,^13^ we obtain different expressions under these different assumptions for the basic staircase design. As seen here and also noted elsewhere, assuming a linear time effect yields a slightly lower variance of the treatment effect estimator than assuming categorical period effects.^14^ A more conservative sample size or power calculation could therefore be conducted by assuming categorical rather than linear period effects.

We have derived explicit expressions for the variance of the treatment effect estimator for the basic staircase design and an expression for general staircase designs in terms of the matrices corresponding to model (2) at the cluster-period mean level. These expressions can be used in sample size and power calculations for staircase designs. While we would have preferred an explicit expression for general staircase designs, further simplification of expression (3) would require inverting the matrix $\sum_{s=1}^{S}{Z_{s}}^{T}{V_{*}}^{-1}Z_{s}$ which can have a complex form depending on the trial characteristics, the assumed intracluster correlation structure, and the assumed form for the time effects. Assuming categorical period effects, for example, pre-multiplication by ${Z_{s}}^{T}$ and post-multiplication by $Z_{s}$ places the elements of ${V_{*}}^{-1}$ into a $\left(S+R_{0}+R_{1}-1)\times (S+R_{0}+R_{1}-1\right)$ matrix in the location corresponding to the observed periods, with staggered overlap in the location of the elements of ${V_{*}}^{-1}$ over the *S* sequences. The summation over all *S* sequences then adds the elements from these partially overlapping matrices, yielding $\left(R_{0}+R_{1}+1\right)$ diagonal bands of nonzero elements with the remaining diagonals made up of zeros. For the basic staircase, this yielded a tridiagonal matrix for which there are many papers covering representations of its inverse.^27^ Even still, the elements of the inverse of a tridiagonal matrix are obtained through recurrence relations, making a simple analytical form difficult.^28^ We instead used recent results on the summation of the elements in a row of the inverse^20^ to obtain a relatively simple analytical form for $\sum_{s=1}^{S}Z_{s}(\sum_{s=1}^{S}{Z_{s}}^{T}{V_{*}}^{-1}Z_{s}{)}^{-1}\sum_{s=1}^{S}{Z_{s}}^{T}$ within expression (3), from which the explicit expression (6) followed. For designs with more than one pre- and/or post-switch measurement period, it is less clear how to obtain a general form for the inverse of the matrix $\sum_{s=1}^{S}{Z_{s}}^{T}{V_{*}}^{-1}Z_{s}$ or summations of certain sets of elements from this inverse.

The variance of the treatment effect estimator expression we derived for general staircase designs assumes that the observed periods in each sequence follow the same schedule of control and intervention periods, simply shifted in time; there may be a benefit to sequences following different schedules. For instance, Kasza et al.^11^ considered a staircase-like design where sequences commencing data collection earlier in the trial had more intervention periods than control periods and sequences appearing later in the trial had more control periods than intervention periods. These types of designs also naturally arise if a staircase design with more than one pre- and/or post-switch period is considered as a subset of the cluster-periods in a complete stepped wedge: since a standard stepped wedge has just one all-control period at the start and one all-intervention period at the end, any staircase design other than a basic staircase taken as a subset of the stepped wedge will have some of its sequences truncated at the edges of the stepped wedge. For example, this occurs when specifying a staircase design with the SteppedPower R package^23^: it can be obtained by specifying an incomplete stepped wedge design, however, the sequences at the edges of the design schematic may be truncated.

Interestingly, the cluster-period measurements in the first and last periods are not used in the treatment effect estimator obtained from generalised least squares under an assumption of categorical period effects because the treatment and time effects cannot be separated. For example, the treatment effect estimator for a three-sequence basic staircase design matches that for the three-sequence dog-leg design, in which the first and third sequences contain only one period each, an intervention and control period, respectively.^29^ However, under the stronger assumption of a linear time effect, the cluster-period measurements in the first and last periods *do* contribute to the treatment effect estimate, albeit with less weight than measurements from the intermediate cluster-periods of the trial.

The formulae we derive in this paper pertain to the generalised least squares estimator of the treatment effect, are based on asymptotic properties, and make the typical assumption of known variance and correlation parameters. The Type I error rate may be elevated and actual power may be lower than the theoretical power calculated with these formulae, particularly if the trial includes few clusters because there are few degrees of freedom for estimating the variance components.^30^ Further, correlation parameter estimates arising from such trials ought to be treated with a degree of caution. When planning trials, simulation studies can be conducted to provide further insight into the performance of these estimators.

In this paper we have considered the staircase design in the context of linear mixed models for outcomes. Work is currently underway on the efficiency of staircase designs as compared with alternative incomplete designs and with complete designs run over fewer time periods. Future work will consider the properties of these designs when outcomes are modelled using non-linear link functions and estimation of the treatment effect is via generalised estimating equations. We expect that several of our theoretical results will provide useful starting points for these investigations. We also plan to investigate the properties of other types of staircase designs beyond the basic staircase in upcoming work. In particular, we intend to examine the efficiency of balanced designs with more than one pre- and post-switch measurement period (i.e., with $R_{0}=R_{1}>1$ ), imbalanced staircase designs (i.e., with $R_{0}\neq R_{1}$ ), staircase designs with a transition period, and more general staircase designs for which the sequences may follow different schedules.

## Supplemental Material

sj-pdf-1-smm-10.1177_09622802231202364 - Supplemental material for The staircase cluster randomised trial design: A pragmatic alternative to the stepped wedge Click here for additional data file. Supplemental material, sj-pdf-1-smm-10.1177_09622802231202364 for The staircase cluster randomised trial design: A pragmatic alternative to the stepped wedge by Kelsey L Grantham, Andrew B Forbes, Richard Hooper and Jessica Kasza in Statistical Methods in Medical Research

## Appendix

### A: Example code for power calculations

This section contains example inputs and/or code to implement power calculations for a basic staircase design with four sequences and two clusters per sequence, SC(4,2,1,1) , a cluster-period size of m=50 , a standardised effect size of 0.375 , assuming a discrete-time decay intracluster correlation structure with within-period ICC $\rho_{0}=0.05$ and cluster autocorrelation r=0.8 . The code for the SteppedPower R package and the inputs for the Shiny CRT calculator specify categorical period effects, and the code for the %CRTFASTGEEPWR SAS macro specifies linear period effects.

#### SteppedPower R package

glsPower(Cl = rep(2,4), mu0 = 0, mu1 = 0.375, sigma = sqrt(0.95), tau = sqrt(0.05),

   AR = 0.8, N = 50, incomplete = 1)

#### %CRTFASTGEEPWR SAS macro

%CRTFASTGEEPWR(alpha = 0.05, m=%str(J(4,1,2)), corr_type = 2,

        alpha0 = 0.05, r0 = 0.8, intervention_effect_type = 1,

        delta = 0.375, period_effect_type = 2,

        beta_period_effects=%str({1,1}), dist=“normal”, phi = 1,

        CP_size_matrix=%str({50 50 0 0 0,

                 0 50 50 0 0,

                 0 0 50 50 0,

                 0 0 0 50 50}),

        DesignPattern=%str({0 1 2 2 2,

                   2 0 1 2 2,

                   2 2 0 1 2,

                   2 2 2 0 1}));

#### Shiny CRT Calculator

**Table:**

**Field**	**Entry**
Trial design	Upload own design:
	0,1,,,
	0,1,,,
	,0,1,,
	,0,1,,
	,,0,1,
	,,0,1,
	,,,0,1
	,,,0,1
	(Uploaded as .csv file)
Sampling structure	Cross-sectional sample
Correlation structure	Discrete time decay
Plot set-up	Cluster size vs. Power
Number of clusters (per sequence)	1
X-axis range: Cluster size (per period)	0–50
Within-period ICC	0.05
Within-period ICC lower extreme	0.01
Within-period ICC upper extreme	0.1
Cluster auto-correlation (CAC)	0.8
Outcome type	Continuous
Mean Difference	0.375
Standard Deviation	1
Variation in treatment effect across clusters	False
Significance level	0.05
