# Supplementary material for: The staircase cluster randomised trial design: A pragmatic alternative to the stepped wedge
Source: Stat Methods Med Res. 2023 Nov 30;33(1):24–41. doi: 10.1177/09622802231202364 (PMC10863363; doi:10.1177/09622802231202364)
Supplement: sj-pdf-1-smm-10.1177_09622802231202364 - Supplemental material for The staircase cluster randomised trial design: A pragmatic alternative to the stepped wedge [file sj-pdf-1-smm-10.1177_09622802231202364.pdf]

Supporting Information

The staircase cluster randomised trial design: a pragmatic alternative to the stepped wedge

Kelsey L. Grantham, Andrew B. Forbes, Richard Hooper, Jessica Kasza

A: Derivation of the variance of the treatment effect estimator, general staircase designs

Letting $\bar{\mathbf{Y}} = \left(\bar{\mathbf{Y}}_{11}^\top, \ldots, \bar{\mathbf{Y}}_{1K}^\top, \ldots, \bar{\mathbf{Y}}_{S1}^\top, \ldots, \bar{\mathbf{Y}}_{SK}^\top\right)^\top$, we can write model (2) in vector form as

$$\begin{aligned}\bar{\mathbf{Y}} &= \mathbf{Z}\boldsymbol{\eta} + \mathbf{CP} + \boldsymbol{\epsilon}\\ &= \begin{pmatrix}\mathbf{Z}_1 & \mathbf{X}_1\\ \vdots & \vdots\\ \mathbf{Z}_1 & \mathbf{X}_1\\ \vdots & \vdots\\ \mathbf{Z}_S & \mathbf{X}_S\\ \vdots & \vdots\\ \mathbf{Z}_S & \mathbf{X}_S\end{pmatrix}\begin{pmatrix}\boldsymbol{\beta}\\ \theta\end{pmatrix} + \begin{pmatrix}\mathbf{CP}_{11}\\ \vdots\\ \mathbf{CP}_{1K}\\ \vdots\\ \mathbf{CP}_{S1}\\ \vdots\\ \mathbf{CP}_{SK}\end{pmatrix} + \begin{pmatrix}\boldsymbol{\epsilon}_{11}\\ \vdots\\ \boldsymbol{\epsilon}_{1K}\\ \vdots\\ \boldsymbol{\epsilon}_{S1}\\ \vdots\\ \boldsymbol{\epsilon}_{SK}\end{pmatrix}\end{aligned}$$

where $\mathbf{Z} = \begin{pmatrix}\mathbf{Z}_1 & \cdots & \mathbf{Z}_S\\ \mathbf{X}_1 & \cdots & \mathbf{X}_S\end{pmatrix}^\top \otimes \mathbf{1}_K$, $\mathbf{X}_s = (X_{ss}, \ldots, X_{s,s+R_0+R_1-1})^\top$ is an $(R_0 + R_1)$-dimensional vector of treatment indicators for sequence s, and $\boldsymbol{\epsilon}_{sk} = (\epsilon_{sks}, \ldots, \epsilon_{sk,s+R_0+R_1-1})^\top$. Since the vector of treatment indicators is common across sequences, we will let $\mathbf{X}_s = \mathbf{X}$.

Let the covariance matrix of $\bar{\mathbf{Y}}$ be $\mathbf{V}$. Then $\mathbf{V}$ is a block-diagonal matrix with $(R_0+R_1)\times(R_0+R_1)$ matrices $\mathbf{V}_*$ on the diagonal. The estimator obtained using generalised least squares is given by $\hat{\boldsymbol{\eta}} = (\mathbf{Z}^\top\mathbf{V}^{-1}\mathbf{Z})^{-1}\mathbf{Z}^\top\mathbf{V}^{-1}\bar{\mathbf{Y}}$ and $cov(\hat{\boldsymbol{\eta}}) = (\mathbf{Z}^\top\mathbf{V}^{-1}\mathbf{Z})^{-1}$. We seek to derive a formula for $var(\hat{\theta})$, the bottom-right element of $cov(\hat{\boldsymbol{\eta}})$. We will first calculate $\mathbf{Z}^\top\mathbf{V}^{-1}\mathbf{Z}$.

We have

$$\begin{aligned}\mathbf{Z}^\top\mathbf{V}^{-1}\mathbf{Z} &= \begin{pmatrix}\mathbf{Z}_1^\top & \cdots & \mathbf{Z}_1^\top & \cdots & \mathbf{Z}_S^\top & \cdots & \mathbf{Z}_S^\top\\ \mathbf{X}^\top & \cdots & \mathbf{X}^\top & \cdots & \mathbf{X}^\top & \cdots & \mathbf{X}^\top\end{pmatrix}\begin{pmatrix}\mathbf{V}_*^{-1} & 0 & \cdots & 0\\ 0 & \mathbf{V}_*^{-1} & & \vdots\\ \vdots & & \ddots & 0\\ 0 & \cdots & 0 & \mathbf{V}_*^{-1}\end{pmatrix}\begin{pmatrix}\mathbf{Z}_1 & \mathbf{X}\\ \vdots & \vdots\\ \mathbf{Z}_1 & \mathbf{X}\\ \vdots & \vdots\\ \mathbf{Z}_S & \mathbf{X}\\ \vdots & \vdots\\ \mathbf{Z}_S & \mathbf{X}\end{pmatrix}\\ &= \begin{pmatrix}K\sum_{s=1}^S \mathbf{Z}_s^\top\mathbf{V}_*^{-1}\mathbf{Z}_s & K\sum_{s=1}^S \mathbf{Z}_s^\top\mathbf{V}_*^{-1}\mathbf{X}\\ K\mathbf{X}^\top\mathbf{V}_*^{-1}\sum_{s=1}^S \mathbf{Z}_s & SK\mathbf{X}^\top\mathbf{V}_*^{-1}\mathbf{X}\end{pmatrix}\\ &= K\begin{pmatrix}\sum_{s=1}^S \mathbf{Z}_s^\top\mathbf{V}_*^{-1}\mathbf{Z}_s & \sum_{s=1}^S \mathbf{Z}_s^\top\mathbf{V}_*^{-1}\mathbf{X}\\ \mathbf{X}^\top\mathbf{V}_*^{-1}\sum_{s=1}^S \mathbf{Z}_s & S\mathbf{X}^\top\mathbf{V}_*^{-1}\mathbf{X}\end{pmatrix}.\end{aligned}$$

Let

$$\begin{pmatrix}\mathbf{A}_{11} & \mathbf{A}_{12}\\ \mathbf{A}_{21} & \mathbf{A}_{22}\end{pmatrix} = \begin{pmatrix}\sum_{s=1}^S \mathbf{Z}_s^\top\mathbf{V}_*^{-1}\mathbf{Z}_s & \sum_{s=1}^S \mathbf{Z}_s^\top\mathbf{V}_*^{-1}\mathbf{X}\\ \mathbf{X}^\top\mathbf{V}_*^{-1}\sum_{s=1}^S \mathbf{Z}_s & S\mathbf{X}^\top\mathbf{V}_*^{-1}\mathbf{X}\end{pmatrix}.$$

Then using the definition of the inverse of a partitioned matrix, the bottom-right element of $(\mathbf{Z}^\top\mathbf{V}^{-1}\mathbf{Z})^{-1}$ is given by $\frac{1}{K}(\mathbf{A}_{22} - \mathbf{A}_{21}\mathbf{A}_{11}^{-1}\mathbf{A}_{12})^{-1}$ and so

$$\begin{aligned} var(\hat{\theta})_{SC} &= \frac{1}{K}\left[S\mathbf{X}^\top\mathbf{V}_*^{-1}\mathbf{X} - \mathbf{X}^\top\mathbf{V}_*^{-1}\sum_{s=1}^{S}\mathbf{Z}_s\left(\sum_{s=1}^{S}\mathbf{Z}_s^\top\mathbf{V}_*^{-1}\mathbf{Z}_s\right)^{-1}\sum_{s=1}^{S}\mathbf{Z}_s^\top\mathbf{V}_*^{-1}\mathbf{X}\right]^{-1} \\ &= \frac{1}{SK}\left[\mathbf{X}^\top\mathbf{V}_*^{-1}\mathbf{X} - \frac{1}{S}\mathbf{X}^\top\mathbf{V}_*^{-1}\sum_{s=1}^{S}\mathbf{Z}_s\left(\sum_{s=1}^{S}\mathbf{Z}_s^\top\mathbf{V}_*^{-1}\mathbf{Z}_s\right)^{-1}\sum_{s=1}^{S}\mathbf{Z}_s^\top\mathbf{V}_*^{-1}\mathbf{X}\right]^{-1}. \end{aligned}$$

B: Derivation of the treatment effect estimator and its variance, four-sequence basic staircase design

B.1 Finding optimal weights, categorical period effects

In Section 3.2.2, we obtain the following general form for the treatment effect estimator:

$$\hat{\theta} = w_1\left(\bar{Y}_{12} - \bar{Y}_{22}\right) + w_2\left(\bar{Y}_{23} - \bar{Y}_{33}\right) + w_3\left(\bar{Y}_{34} - \bar{Y}_{44}\right)$$

where $w_1 + w_2 + w_3 = 1$, which has variance

$$var(\hat{\theta}) = 2a\left[w_1^2 + w_2^2 + w_3^2 - \psi(w_1w_2 + w_2w_3)\right].$$

For the four-sequence basic staircase design, we can find the weights that give the lowest variance by minimising $a\left[w_1^2 + w_2^2 + w_3^2 - \psi\left(w_1w_2 + w_2w_3\right)\right]$ subject to the constraint $w_1 + w_2 + w_3 - 1 = 0$ using Lagrange multiplier equations.

Let $\mathcal{L}(w_1, w_2, w_3, \lambda) = f(w_1, w_2, w_3) + \lambda \cdot g(w_1, w_2, w_3)$ where $f(w_1, w_2, w_3) = a\left[w_1^2 + w_2^2 + w_3^2 - \psi\left(w_1w_2 + w_2w_3\right)\right]$ and $g(w_1, w_2, w_3) = w_1 + w_2 + w_3 - 1$. Then we solve $\nabla_{w_1,w_2,w_3,\lambda}\mathcal{L}(w_1, w_2, w_3, \lambda) = 0$, which yields the following system of equations:

$$\begin{aligned} a\left(2w_1 - \psi w_2\right) + \lambda &= 0 \quad (1) \\ a\left[2w_2 - \psi\left(w_1 + w_3\right)\right] + \lambda &= 0 \quad (2) \\ a\left(2w_3 - \psi w_2\right) + \lambda &= 0 \quad (3) \\ w_1 + w_2 + w_3 - 1 &= 0 \quad (4) \end{aligned}$$

B.1.1 Approach 1: Manually solve system of equations

Subtracting (3) from (1) gives: $w_1 = w_3$, and subtracting (3) from (2) together with the result that $w_1 = w_3$ gives: $w_2 = \frac{2+2\psi}{2+\psi}w_3$. Then plugging these results into (4) gives: $w_1 = \frac{2+\psi}{2(3+2\psi)} = w_3$ and $w_2 = \frac{1+\psi}{3+2\psi}$.

B.1.2 Approach 2: Matrix inversion

Another way to solve the system of Lagrange multiplier equations is by expressing the problem in vector and matrix form. Then the vector of weights can be written as

$$\begin{aligned} \begin{pmatrix} w_1 \\ w_2 \\ w_3 \end{pmatrix} &= \frac{1}{a}\begin{pmatrix} 2 & -\psi & 0 \\ -\psi & 2 & -\psi \\ 0 & -\psi & 2 \end{pmatrix}^{-1}\begin{pmatrix} -\lambda \\ -\lambda \\ -\lambda \end{pmatrix} \\ &= -\lambda \cdot \Sigma^{-1}\mathbf{1} \end{aligned}$$

where $\mathbf{1}$ is a column vector of ones. Since the weights must also satisfy the condition $w_1 + w_2 + w_3 = 1$, then

$$1 = \mathbf{1}^\top\begin{pmatrix} w_1 \\ w_2 \\ w_3 \end{pmatrix} = -\lambda \cdot \mathbf{1}^\top\Sigma^{-1}\mathbf{1}$$

and so $-\lambda = \frac{1}{\mathbf{1}^\top \Sigma^{-1} \mathbf{1}}$. Then the weights can be represented as

$$\begin{pmatrix} w_1 \\ w_2 \\ w_3 \end{pmatrix} = \frac{1}{\mathbf{1}^\top \Sigma^{-1} \mathbf{1}} \begin{pmatrix} s_1 \\ s_2 \\ s_3 \end{pmatrix}$$

where s_i is the sum of the elements in row i of Σ^{-1}. Then since

$$\Sigma^{-1} = \frac{1}{a} \begin{pmatrix} \frac{4-\psi^2}{4(2-\psi^2)} & \frac{2\psi}{4(2-\psi^2)} & \frac{\psi^2}{4(2-\psi^2)} \\ \frac{2\psi}{4(2-\psi^2)} & \frac{4}{4(2-\psi^2)} & \frac{2\psi}{4(2-\psi^2)} \\ \frac{\psi^2}{4(2-\psi^2)} & \frac{2\psi}{4(2-\psi^2)} & \frac{4-\psi^2}{4(2-\psi^2)} \end{pmatrix},$$

the optimal weights can be obtained using the row sums and total sum of the elements of Σ^{-1}:

$$\begin{pmatrix} w_1 \\ w_2 \\ w_3 \end{pmatrix} = \frac{a(2-\psi^2)}{3+2\psi} \begin{pmatrix} \frac{2+\psi}{2a(2-\psi^2)} \\ \frac{2+2\psi}{2a(2-\psi^2)} \\ \frac{2+\psi}{2a(2-\psi^2)} \end{pmatrix} = \begin{pmatrix} \frac{2+\psi}{2(3+2\psi)} \\ \frac{1+\psi}{3+2\psi} \\ \frac{2+\psi}{2(3+2\psi)} \end{pmatrix}.$$

B.2 Linear period effects

Consider again the $SC(4,1,1,1)$ design, a four-sequence basic staircase design with one cluster per sequence. We begin with the general estimator for θ, expressed as a linear combination of the weighted cluster-period means:

$$\hat{\theta} = w_{11}\bar{Y}_{11} + w_{12}\bar{Y}_{12} + w_{22}\bar{Y}_{22} + w_{23}\bar{Y}_{23} + w_{33}\bar{Y}_{33} + w_{34}\bar{Y}_{34} + w_{44}\bar{Y}_{44} + w_{45}\bar{Y}_{45}.$$

Then under model (2) and assuming *linear* period effects such that $\mathbf{Z}_{st}\boldsymbol{\beta} = \beta_1 + \beta_2 t$, $\hat{\theta}$ has expectation

$$\begin{aligned} E\left(\hat{\theta}\right) &= (w_{12} + w_{23} + w_{34} + w_{45})\,\theta + (w_{11} + w_{12} + w_{22} + w_{23} + w_{33} + w_{34} + w_{44} + w_{45})\,\beta_1 \\ &\quad + \left[w_{11} + 2\,(w_{12} + w_{22}) + 3\,(w_{23} + w_{33}) + 4\,(w_{34} + w_{44}) + 5w_{45}\right]\beta_2. \end{aligned}$$

Since we want $\hat{\theta}$ to be an unbiased estimator of θ, the following conditions must hold:

(i) $w_{11} + w_{12} + w_{22} + w_{23} + w_{33} + w_{34} + w_{44} + w_{45} = 0$

(ii) $w_{11} + 2\,(w_{12} + w_{22}) + 3\,(w_{23} + w_{33}) + 4\,(w_{34} + w_{44}) + 5w_{45} = 0$

(iii) $w_{12} + w_{23} + w_{34} + w_{45} = 1$

Conditions (i) and (iii) imply that: $w_{11} + w_{22} + w_{33} + w_{44} = -1$. Furthermore, according to Bowden et al (2021), a basic staircase design is skew-symmetric and model (2) is a centrosymmetric model. Therefore, the most efficient estimator for the treatment effect has a centrosymmetric vector of weights on the cluster-period means, i.e. $w_{st} = -w_{S+1-s,T+1-t}$. Then we can rewrite the treatment effect estimator expression in terms of four unique weights, letting $w_1 = w_{12} = -w_{44}$, $w_2 = w_{23} = -w_{33}$, $w_3 = w_{34} = -w_{22}$, and $w_4 = w_{45} = -w_{11}$:

$$\hat{\theta} = w_1\left(\bar{Y}_{12} - \bar{Y}_{44}\right) + w_2\left(\bar{Y}_{23} - \bar{Y}_{33}\right) + w_3\left(\bar{Y}_{34} - \bar{Y}_{22}\right) + w_4\left(\bar{Y}_{45} - \bar{Y}_{11}\right)$$

where $w_1 + w_2 + w_3 + w_4 = 1$ and $-2w_1 + 2w_3 + 4w_4 = 0$, from conditions (iii) and (ii), respectively.

Note that unlike when categorical period effects are assumed, all cluster-period means contribute to the treatment effect estimate when linear period effects are assumed.

Then the variance of the treatment effect estimator can be represented as

$$\begin{aligned} var(\hat{\theta}) &= 2a\left(w_1^2 + w_2^2 + w_3^2 + w_4^2\right) - 2b\,(2w_1w_4 + 2w_2w_3) \\ &= 2a\left[w_1^2 + w_2^2 + w_3^2 + w_4^2 - 2\psi\,(w_1w_4 + w_2w_3)\right]. \end{aligned}$$

We can find the optimal weights by using the method of Lagrange multipliers to minimise the expression $a\left[w_1^2 + w_2^2 + w_3^2 + w_4^2 - 2\psi\,(w_1w_4 + w_2w_3)\right]$ subject to the constraints $w_1 + w_2 + w_3 + w_4 - 1 = 0$ and $-2w_1 + 2w_3 + 4w_4 = 0$.

Let $\mathcal{L}(w_1, w_2, w_3, w_4, \lambda_1, \lambda_2) = f(w_1, w_2, w_3, w_4) - [\lambda_1 \cdot g_1(w_1, w_3, w_4) + \lambda_2 \cdot g_2(w_1, w_2, w_3, w_4)]$, where $f(w_1, w_2, w_3, w_4) = a\left[w_1^2 + w_2^2 + w_3^2 + w_4^2 - 2\psi\left(w_1 w_4 + w_2 w_3\right)\right]$, $g_1(w_1, w_3, w_4) = -2w_1 + 2w_3 + 4w_4$, and $g_2(w_1, w_2, w_3, w_4) = w_1 + w_2 + w_3 + w_4 - 1$. Then we solve $\nabla_{w_1,w_2,w_3,w_4,\lambda_1,\lambda_2}\mathcal{L}(w_1, w_2, w_3, w_4, \lambda_1, \lambda_2) = 0$, which yields the following system of equations:

$$\begin{aligned} a\left(2w_1 - 2\psi w_4\right) + 2\lambda_1 - \lambda_2 &= 0 \quad (1) \\ a\left(2w_2 - 2\psi w_3\right) - \lambda_2 &= 0 \quad (2) \\ a\left(2w_3 - 2\psi w_2\right) - 2\lambda_1 - \lambda_2 &= 0 \quad (3) \\ a\left(2w_4 - 2\psi w_1\right) - 4\lambda_1 - \lambda_2 &= 0 \quad (4) \\ -2w_1 + 2w_3 + 4w_4 &= 0 \quad (5) \\ w_1 + w_2 + w_3 + w_4 - 1 &= 0 \quad (6) \end{aligned}$$

Subtracting (4) from (1) gives: $w_1 = w_4 - \frac{3}{a(1+\psi)}\lambda_1$, subtracting (3) from (2) gives: $w_2 = w_3 - \frac{1}{a(1+\psi)}\lambda_1$. Then (3) becomes $(3)^* = 2a(1-\psi)w_3 - \frac{2}{1+\psi}\lambda_1 - \lambda_2 = 0$ and (4) becomes $(4)^* = 2a(1-\psi)w_4 - \frac{2(2-\psi)}{1+\psi}\lambda_1 - \lambda_2 = 0$. Then subtracting $(4)^*$ from $(3)^*$ gives: $w_3 = w_4 - \frac{1}{a(1+\psi)}\lambda_1$. Plugging the previous results into (5) gives: $w_4 = -\frac{1}{a(1+\psi)}\lambda_1$ and into (6) gives: $w_4 = \frac{1}{4} + \frac{3}{2a(1+\psi)}\lambda_1$. Equating these results gives: $\lambda_1 = -\frac{a(1+\psi)}{10}$. Then plugging this result into each of the forms for each w_i, we obtain the following weights:

$$w_1 = \frac{4}{10}, w_2 = \frac{3}{10}, w_3 = \frac{2}{10}, w_4 = \frac{1}{10}.$$

Unlike when categorical period effects are assumed, these weights do not depend on the correlation between cluster-period means, ψ. Then the treatment effect estimator can be represented as

$$\begin{aligned} \hat{\theta} &= \frac{1}{10}\left[4\left(\bar{Y}_{12} - \bar{Y}_{44}\right) + 3\left(\bar{Y}_{23} - \bar{Y}_{33}\right) + 2\left(\bar{Y}_{34} - \bar{Y}_{22}\right) + \left(\bar{Y}_{45} - \bar{Y}_{11}\right)\right] \\ &= \frac{1}{10}\left[4\bar{Y}_{12} + 3\bar{Y}_{23} + 2\bar{Y}_{34} + \bar{Y}_{45} - \left(\bar{Y}_{11} + 2\bar{Y}_{22} + 3\bar{Y}_{33} + 4\bar{Y}_{44}\right)\right]. \end{aligned}$$

Each of the cluster-period means under a particular treatment condition have different weights: the cluster-period means under the intervention condition have decreasing magnitude, and the weights on the cluster-period means under the control condition have increasing magnitude (and opposite sign to those under the intervention condition), with progression in time across the periods of the trial.

Finally, the variance of the treatment effect estimator obtained by plugging the weights back into the variance expression and simplifying gives

$$var(\hat{\theta})_{SC(4,1,1,1),lin} = \frac{a\left(3 - 2\psi\right)}{5}.$$

If K clusters were randomised to each sequence, then the variance would simply be reduced by a factor of $1/K$:

$$var(\hat{\theta})_{SC(4,K,1,1),lin} = \frac{a\left(3 - 2\psi\right)}{5K}.$$

C Derivation of the variance of the treatment effect estimator, S-sequence basic staircase design

C.1 Categorical period effects

Following the approach outlined in Section 3.2 but for an S-sequence basic staircase design, we can write the treatment effect estimator as

$$\hat{\theta} = \sum_{s=1}^{S}\sum_{t=s}^{s+1} w_{st}\bar{Y}_{st},$$

which has expectation

$$E\left(\hat{\theta}\right) = \left(\sum_{s=1}^{S-1} w_{s,s+1}\right)\theta + w_{11}\beta_1 + \sum_{t=2}^{S}\sum_{s=t-1}^{t} w_{st}\beta_t + w_{S,S+1}\beta_{S+1}.$$

And since we want $\hat{\theta}$ to be an unbiased estimator of θ, the following conditions must hold:

(i) $w_{11} = 0$ and $w_{S,S+1} = 0$

(ii) $\sum_{s=t-1}^{t} w_{st} = 0$ for $t = 2, \ldots, S$

(iii) $\sum_{s=1}^{S-1} w_{s,s+1} = 1$.

As seen in the four-sequence case, (i) implies that the outcomes from the cluster-periods in the first and last periods have weights of zero and therefore do not contribute to the treatment effect estimate, and (ii) implies that the cluster-periods measured in the same period receive weights that are equal in magnitude but opposite signs. Then letting $w_s = w_{s,s+1} = -w_{s+1,s+1}$ for all $s = 1, \ldots, S-1$, the treatment effect estimator $\hat{\theta}$ can be written in simplified form as

$$\hat{\theta} = \sum_{s=1}^{S-1} w_s \left(\bar{Y}_{s,s+1} - \bar{Y}_{s+1,s+1}\right)$$

with the constraint that $\sum_{s=1}^{S-1} w_s = 1$, and the variance of the treatment effect estimator can be represented as

$$var(\hat{\theta}) = 2a \left(\sum_{s=1}^{S-1} w_s^2 - \psi \sum_{s=1}^{S-2} w_s w_{s+1} \right).$$

The optimal weights w_s that give lowest variance can be found by solving the system of $S-1$ Lagrange multiplier equations. A general analytical form for the weights can be obtained by representing the system of equations as a matrix equation. Then the vector of weights can be expressed in terms of the elements of the inverse of an $(S-1) \times (S-1)$ symmetric tridiagonal Toeplitz matrix Σ, where

$$\Sigma \quad = a \begin{pmatrix} 2 & -\psi & 0 & \cdots & 0 \\ -\psi & 2 & -\psi & & \vdots \\ 0 & -\psi & 2 & \ddots & 0 \\ \vdots & & \ddots & \ddots & -\psi \\ 0 & \cdots & 0 & -\psi & 2 \end{pmatrix}.$$

The matrix Σ represents the covariance matrix of "one-dependent random variables", i.e. the differences in cluster-period means: $\left(\bar{Y}_{s,s+1} - \bar{Y}_{s+1,s+1}\right)$, which each have variance $2a$ and covariance $-a\psi(= -b)$ with the differences in adjacent periods (da Fonseca and Petronilho, 2001). Then the optimal weights can be represented as

$$w_i = \frac{s_i}{\mathbf{1}^\top \Sigma^{-1} \mathbf{1}},$$

where $s_i = \frac{1-\psi a(\sigma_{1i}+\sigma_{1,S-i})}{2a(1-\psi)}$ is the sum of row i of Σ^{-1} and $\mathbf{1}^\top \Sigma^{-1} \mathbf{1} = \frac{S-1-2\psi a \cdot s_1}{2a(1-\psi)}$ is the sum of all elements of Σ^{-1}, with $\sigma_{ij} = \left(\Sigma^{-1}\right)_{ij}$. The weights can then be expressed solely in terms of the elements of the first row of Σ^{-1}:

$$\begin{aligned} w_i &= \frac{1 - \psi a \left(\sigma_{1i} + \sigma_{1,S-i}\right)}{S - 1 - 2\psi a \cdot s_1} \\ &= \frac{1 - \psi a \left(\sigma_{1i} + \sigma_{1,S-i}\right)}{S - 1 - \frac{\psi}{1-\psi}\left[1 - \psi a \left(\sigma_{11} + \sigma_{1,S-1}\right)\right]} \end{aligned}$$

where $\sigma_{1i} + \sigma_{1,S-i} = \frac{(-1)^i\left(\alpha_+^{S-i} - \alpha_-^{S-i}\right) + (-1)^{S-i}\left(\alpha_+^i - \alpha_-^i\right)}{\psi a\left(\alpha_+^S - \alpha_-^S\right)}$, and $\alpha_\pm = \frac{1 \pm \sqrt{1-\psi^2}}{-\psi}$.

While the variance of the treatment effect estimator can be obtained by plugging these weights back into the earlier variance expression and simplifying, since we are dealing with "one-dependent random variables", we can exploit the more direct result that the precision of the treatment effect estimator is

simply the sum of all the elements of Σ^{-1} (da Fonseca and Petronilho, 2001):

$$\begin{aligned}
var(\hat{\theta})_{SC(S,1,1,1),cat} &= \frac{1}{\mathbf{1}^\top \Sigma^{-1} \mathbf{1}} = \frac{2a(1-\psi)}{S-1-2\psi a \cdot s_1} \\
&= \frac{2a(1-\psi)}{S-1-\frac{\psi}{1-\psi}\left[1-\psi a\left(\sigma_{11}+\sigma_{1,S-1}\right)\right]} \\
&= \frac{2a(1-\psi)^2}{S(1-\psi)-1+\psi\cdot\frac{-\left(\alpha_+^{S-1}-\alpha_-^{S-1}\right)+(-1)^{S-1}(\alpha_+-\alpha_-)}{\alpha_+^S-\alpha_-^S}} \\
&= \frac{2a(1-\psi)^2}{S(1-\psi)+\sqrt{1-\psi^2}\cdot\frac{2\psi^S-\left(1+\sqrt{1-\psi^2}\right)^S-\left(1-\sqrt{1-\psi^2}\right)^S}{\left(1+\sqrt{1-\psi^2}\right)^S-\left(1-\sqrt{1-\psi^2}\right)^S}} \\
&= \frac{2a(1-\psi)^2}{S(1-\psi)-\sqrt{1-\psi^2}\frac{\left(1+\sqrt{1-\psi^2}\right)^S-\psi^S}{\left(1+\sqrt{1-\psi^2}\right)^S+\psi^S}}.
\end{aligned}$$

If K clusters were randomised to each of the S sequences of the design, then the variance of the treatment effect estimator would simply be reduced by a factor of $1/K$, so that

$$var(\hat{\theta})_{SC(S,K,1,1),cat} = \frac{2a(1-\psi)^2}{K\left[S(1-\psi)-\sqrt{1-\psi^2}\frac{\left(1+\sqrt{1-\psi^2}\right)^S-\psi^S}{\left(1+\sqrt{1-\psi^2}\right)^S+\psi^S}\right]}.$$

C.2 Categorical period effects, using expression (3)

C.2.1 Setup

We seek to derive an analytical variance expression for the basic staircase design, with $R_0 = R_1 = 1$. Then $\mathbf{X}^\top = (0,1)$ and the covariance matrix for a single cluster, assumed common across clusters, is of the form

$$\mathbf{V}_* = \begin{pmatrix} a & b \\ b & a \end{pmatrix}$$

and so

$$\mathbf{V}_*^{-1} = \begin{pmatrix} \frac{a}{a^2-b^2} & \frac{-b}{a^2-b^2} \\ \frac{-b}{a^2-b^2} & \frac{a}{a^2-b^2} \end{pmatrix} = \frac{b}{a^2-b^2}\begin{pmatrix} \frac{a}{b} & -1 \\ -1 & \frac{a}{b} \end{pmatrix}.$$

Note that with just two periods of measurement per cluster, the block-exchangeable and discrete-time correlation decay intracluster correlation structures are equivalent.

C.2.2 Categorical period effects

Consider a basic staircase design with S unique treatment sequences. Assuming categorical period effects, the matrices encoding the time effects, $\mathbf{Z}_s, s = 1, \ldots, S$, are $2 \times (S+1)$-dimensional matrices comprised entirely of zeroes except for a 2×2 identity matrix starting in column s:

$$\mathbf{Z}_1 = \begin{pmatrix} 1 & 0 & 0 & \cdots & 0 \\ 0 & 1 & 0 & \cdots & 0 \end{pmatrix}, \quad \mathbf{Z}_2 = \begin{pmatrix} 0 & 1 & 0 & \cdots & 0 \\ 0 & 0 & 1 & \cdots & 0 \end{pmatrix}, \quad \cdots, \quad \mathbf{Z}_S = \begin{pmatrix} 0 & 0 & \cdots & 1 & 0 \\ 0 & 0 & \cdots & 0 & 1 \end{pmatrix}$$

and so

$$\sum_{s=1}^S \mathbf{Z}_s^\top \mathbf{V}_*^{-1} \mathbf{Z}_s = \frac{b}{a^2-b^2}\begin{pmatrix} \frac{a}{b} & -1 & \cdots & 0 & 0 \\ -1 & \frac{2a}{b} & \cdots & 0 & 0 \\ \vdots & \vdots & \ddots & \vdots & \vdots \\ 0 & 0 & \cdots & \frac{2a}{b} & -1 \\ 0 & 0 & \cdots & -1 & \frac{a}{b} \end{pmatrix} = \frac{b}{a^2-b^2}\mathbf{Q}.$$

Note that $\mathbf{Q}$ is a tridiagonal matrix. Now consider

$$\sum_{s=1}^{S} \mathbf{Z}_s \left(\sum_{s=1}^{S} \mathbf{Z}_s^\top \mathbf{V}_*^{-1} \mathbf{Z}_s \right)^{-1} \sum_{s=1}^{S} \mathbf{Z}_s^\top = \frac{a^2 - b^2}{b} \sum_{s=1}^{S} \mathbf{Z}_s \mathbf{Q}^{-1} \sum_{s=1}^{S} \mathbf{Z}_s^\top.$$

Since

$$\sum_{s=1}^{S} \mathbf{Z}_s = \begin{pmatrix} 1 & 1 & \cdots & 1 & 0 \\ 0 & 1 & \cdots & 1 & 1 \end{pmatrix},$$

then

$$\sum_{s=1}^{S} \mathbf{Z}_s \mathbf{Q}^{-1} \sum_{s=1}^{S} \mathbf{Z}_s^\top = \begin{pmatrix} \sum_{i=1}^{S} \sum_{j=1}^{S} \mathbf{Q}_{ij}^{-1} & \sum_{i=1}^{S} \sum_{j=2}^{S+1} \mathbf{Q}_{ij}^{-1} \\ \sum_{i=2}^{S+1} \sum_{j=1}^{S} \mathbf{Q}_{ij}^{-1} & \sum_{i=2}^{S+1} \sum_{j=2}^{S+1} \mathbf{Q}_{ij}^{-1} \end{pmatrix}$$

Since $\mathbf{Q}$ is a symmetric and persymmetric matrix (symmetric about its main and secondary diagonals), $\mathbf{Q}^{-1}$ is also symmetric and persymmetric (Roebuck and Barnett, 1978). Then $\mathbf{Q}_{1S}^{-1} = \mathbf{Q}_{2,(S+1)}^{-1}$, $\mathbf{Q}_{1,(S-1)}^{-1} = \mathbf{Q}_{3,(S+1)}^{-1}$ and $\mathbf{Q}_{12}^{-1} = \mathbf{Q}_{S,(S+1)}^{-1}$ so then $\sum_{i=1}^{S} \sum_{j=1}^{S} \mathbf{Q}_{ij}^{-1} = \sum_{i=2}^{S} \sum_{j=1}^{S+1} \mathbf{Q}_{ij}^{-1} + \mathbf{Q}_{11}^{-1}$. Similarly, we can write the other elements of $\sum_{s=1}^{S} \mathbf{Z}_s \mathbf{Q}^{-1} \sum_{s=1}^{S} \mathbf{Z}_s^\top$ in terms of the sum of the elements of the inner rows and the element in the relevant corner:

$$\begin{aligned} \sum_{s=1}^{S} \mathbf{Z}_s \mathbf{Q}^{-1} \sum_{s=1}^{S} \mathbf{Z}_s^\top &= \begin{pmatrix} \sum_{i=2}^{S} \sum_{j=1}^{S+1} \mathbf{Q}_{ij}^{-1} + \mathbf{Q}_{11}^{-1} & \sum_{i=2}^{S} \sum_{j=1}^{S+1} \mathbf{Q}_{ij}^{-1} + \mathbf{Q}_{1,(S+1)}^{-1} \\ \sum_{i=2}^{S} \sum_{j=1}^{S+1} \mathbf{Q}_{ij}^{-1} + \mathbf{Q}_{(S+1),1}^{-1} & \sum_{i=2}^{S} \sum_{j=1}^{S+1} \mathbf{Q}_{ij}^{-1} + \mathbf{Q}_{(S+1),(S+1)}^{-1} \end{pmatrix} \\ &= \begin{pmatrix} \Omega + \mathbf{Q}_{11}^{-1} & \Omega + \mathbf{Q}_{1,(S+1)}^{-1} \\ \Omega + \mathbf{Q}_{(S+1),1}^{-1} & \Omega + \mathbf{Q}_{(S+1),(S+1)}^{-1} \end{pmatrix} \end{aligned}$$

Tan (2019) provided some useful results pertaining to the inverse of real symmetric $n \times n$ tridiagonal matrices of the form

$$\begin{pmatrix} d & -b & \cdots & 0 & 0 \\ -b & c & \cdots & 0 & 0 \\ \vdots & \vdots & \ddots & \vdots & \vdots \\ 0 & 0 & \cdots & c & -b \\ 0 & 0 & \cdots & -b & d \end{pmatrix},$$

where the first and last diagonal elements differ from the remaining diagonal elements. The results we will use below hold when the inner diagonal element $c \neq 2$. In our case, $d = \frac{a}{b}$, $b = 1$ and $c = 2d = \frac{2a}{b}$, and so $c \neq 2$ since $a \neq b$. For simplicity of notation, we will let $d = \frac{a}{b}$ and substitute a and b back in at the end of the derivation.

The elements of the $(S+1) \times (S+1)$ tridiagonal matrix $\mathbf{Q}^{-1}$ can be represented by $\mathbf{Q}_{ij}^{-1} = u_i v_j$ for $i \leq j$ where

$$v_i = \kappa_{S+1-i}/\kappa \quad u_i = \kappa_{i-1}/\kappa_0 \quad (i = 1, \ldots, S+1)$$

where κ_i, κ and κ_0 can be represented by

$$\begin{aligned} \kappa_i &= \sqrt{d^2 - 1} \left[\left(d + \sqrt{d^2 - 1} \right)^i + \left(d - \sqrt{d^2 - 1} \right)^i \right] \\ \kappa &= \left(d^2 - 1 \right) \left[\left(d + \sqrt{d^2 - 1} \right)^S - \left(d - \sqrt{d^2 - 1} \right)^S \right] \\ \kappa_0 &= 2\sqrt{d^2 - 1} \end{aligned}$$

Then

$$\begin{aligned} \mathbf{Q}_{11}^{-1} = u_1 v_1 = \frac{\kappa_S}{\kappa} = u_{S+1} v_{S+1} = \mathbf{Q}_{(S+1),(S+1)}^{-1} \\ \mathbf{Q}_{1,(S+1)}^{-1} = u_1 v_{S+1} = \frac{\kappa_0}{\kappa} = \mathbf{Q}_{(S+1),1}^{-1} \end{aligned}$$

The sum of the elements of the ith row of $\mathbf{Q}^{-1}$ can be represented by

$$\sum_{j=1}^{S+1} \mathbf{Q}_{ij}^{-1} = \frac{1}{2(d-1)} + \frac{1}{2}\left(v_i + v_{S+2-i}\right)$$

We can then represent Ω, the sum of the elements of the inner rows of $\mathbf{Q}^{-1}$, by

$$\begin{aligned}
\Omega &= \sum_{i=2}^{S} \sum_{j=1}^{S+1} \mathbf{Q}_{ij}^{-1} \\
&= \sum_{i=2}^{S} \left[\frac{1}{2(d-1)} + \frac{1}{2}\left(v_i + v_{S+2-i}\right)\right] \\
&= \frac{S-1}{2(d-1)} + \frac{1}{2}\sum_{i=2}^{S}\left(v_i + v_{S+2-i}\right) \\
&= \frac{S-1}{2(d-1)} + \sum_{i=2}^{S} v_i \\
&= \frac{S-1}{2(d-1)} + \sum_{i=2}^{S} \frac{\kappa_{S+1-i}}{\kappa} \\
&= \frac{S-1}{2(d-1)} + \sum_{i=1}^{S-1} \frac{\kappa_i}{\kappa}.
\end{aligned}$$

$$\begin{aligned}
\sum_{i=1}^{S-1} \kappa_i &= \sqrt{d^2-1}\left[\sum_{i=1}^{S-1}(d+\sqrt{d^2-1})^i + \sum_{i=1}^{S-1}(d-\sqrt{d^2-1})^i\right] \\
&= \sqrt{d^2-1}\left[\frac{(d+\sqrt{d^2-1})-(d+\sqrt{d^2-1})^S}{1-(d+\sqrt{d^2-1})} + \frac{(d-\sqrt{d^2-1})-(d-\sqrt{d^2-1})^S}{1-(d-\sqrt{d^2-1})}\right] \\
&= \frac{-\sqrt{d^2-1}}{2(d-1)}\left[2(d-1) + (d-1-\sqrt{d^2-1})(d+\sqrt{d^2-1})^S + (d-1+\sqrt{d^2-1})(d-\sqrt{d^2-1})^S\right] \\
&= \frac{-\sqrt{d^2-1}}{2(d-1)}\Big[2(d-1) + (d-1)(d+\sqrt{d^2-1})^S + (d-1)(d-\sqrt{d^2-1})^S \\
&\qquad - \sqrt{d^2-1}(d+\sqrt{d^2-1})^S + \sqrt{d^2-1}(d-\sqrt{d^2-1})^S\Big]
\end{aligned}$$

and so

$$\begin{aligned}
\Omega &= \frac{S-1}{2(d-1)} + \sum_{i=1}^{S-1} \frac{\kappa_i}{\kappa} \\
&= \frac{S - \frac{\sqrt{d^2-1}\left[2(d-1)+(d-1)(d+\sqrt{d^2-1})^S+(d-1)(d-\sqrt{d^2-1})^S\right]}{(d^2-1)\left[(d+\sqrt{d^2-1})^S-(d-\sqrt{d^2-1})^S\right]}}{2(d-1)} \\
&= \frac{S - \frac{\sqrt{d^2-1}\left[2+(d+\sqrt{d^2-1})^S+(d-\sqrt{d^2-1})^S\right]}{(d+1)\left[(d+\sqrt{d^2-1})^S-(d-\sqrt{d^2-1})^S\right]}}{2(d-1)}
\end{aligned}$$

$$\begin{aligned}
\Omega + \mathbf{Q}^{-1}_{11} &= \Omega + \mathbf{Q}^{-1}_{(S+1),(S+1)} \\
&= \Omega + \frac{\kappa_S}{\kappa} \\
&= \Omega + \frac{\sqrt{d^2-1}\left[(d+\sqrt{d^2-1})^S + (d-\sqrt{d^2-1})^S\right]}{(d^2-1)\left[(d+\sqrt{d^2-1})^S - (d-\sqrt{d^2-1})^S\right]} \\
&= \frac{S + \frac{\sqrt{d^2-1}\left[-2+(d+\sqrt{d^2-1})^S+(d-\sqrt{d^2-1})^S\right]}{(d+1)\left[(d+\sqrt{d^2-1})^S-(d-\sqrt{d^2-1})^S\right]}}{2(d-1)} \\
\Omega + \mathbf{Q}^{-1}_{1,(S+1)} &= \Omega + \mathbf{Q}^{-1}_{(S+1),1} \\
&= \Omega + \frac{\kappa_0}{\kappa} \\
&= \Omega + \frac{2\sqrt{d^2-1}}{(d^2-1)\left[(d+\sqrt{d^2-1})^S - (d-\sqrt{d^2-1})^S\right]} \\
&= \frac{S - \frac{\sqrt{d^2-1}\left[-2+(d+\sqrt{d^2-1})^S+(d-\sqrt{d^2-1})^S\right]}{(d+1)\left[(d+\sqrt{d^2-1})^S-(d-\sqrt{d^2-1})^S\right]}}{2(d-1)}
\end{aligned}$$

and so

$$\sum_{s=1}^{S} \mathbf{Z}_s \mathbf{Q}^{-1} \sum_{s=1}^{S} \mathbf{Z}_s^\top = \begin{pmatrix} \frac{S+\Phi}{2(d-1)} & \frac{S-\Phi}{2(d-1)} \\ \frac{S-\Phi}{2(d-1)} & \frac{S+\Phi}{2(d-1)} \end{pmatrix}$$

where

$$\Phi = \frac{\sqrt{d^2-1}\left[-2 + (d+\sqrt{d^2-1})^S + (d-\sqrt{d^2-1})^S\right]}{(d+1)\left[(d+\sqrt{d^2-1})^S - (d-\sqrt{d^2-1})^S\right]}.$$

Then

$$\begin{aligned}
\mathbf{X}^\top \mathbf{V}_*^{-1} \sum_{s=1}^{S} \mathbf{Z}_s \mathbf{Q}^{-1} \sum_{s=1}^{S} \mathbf{Z}_s^\top \mathbf{V}_*^{-1} \mathbf{X} &= \frac{b}{a^2-b^2} \cdot \begin{pmatrix} -1 & d \end{pmatrix} \begin{pmatrix} \frac{S+\Phi}{2(d-1)} & \frac{S-\Phi}{2(d-1)} \\ \frac{S-\Phi}{2(d-1)} & \frac{S+\Phi}{2(d-1)} \end{pmatrix} \begin{pmatrix} -1 \\ d \end{pmatrix} \\
&= \frac{b}{a^2-b^2} \cdot \left[(d^2+1) \cdot \frac{S+\Phi}{2(d-1)} - 2d \cdot \frac{S-\Phi}{2(d-1)}\right] \\
&= \frac{b}{a^2-b^2} \cdot \left[\frac{S(d-1)^2 + (d+1)^2\Phi}{2(d-1)}\right]
\end{aligned}$$

and

$$\mathbf{X}^\top \mathbf{V}_*^{-1} \mathbf{X} = \frac{b}{a^2-b^2} \begin{pmatrix} 0 & 1 \end{pmatrix} \begin{pmatrix} d & -1 \\ -1 & d \end{pmatrix} \begin{pmatrix} 0 \\ 1 \end{pmatrix} = \frac{b}{a^2-b^2} \cdot d$$

so then the variance of the treatment effect estimator is given by

$$\begin{aligned}
var(\hat{\theta})_{SC(S,K,1,1),cat} &= \frac{1}{K}\left[S\mathbf{X}^\top\mathbf{V}_*^{-1}\mathbf{X} - \mathbf{X}^\top\mathbf{V}_*^{-1}\sum_{s=1}^{S}\mathbf{Z}_s\left(\sum_{s=1}^{S}\mathbf{Z}_s^\top\mathbf{V}_*^{-1}\mathbf{Z}_s\right)^{-1}\sum_{s=1}^{S}\mathbf{Z}_s^\top\mathbf{V}_*^{-1}\mathbf{X}\right]^{-1} \\
&= \frac{1}{K}\left\{\frac{b}{a^2-b^2}\left[Sd - \frac{S(d-1)^2+(d+1)^2\Phi}{2(d-1)}\right]\right\}^{-1} \\
&= \frac{1}{K}\left\{\frac{b}{a^2-b^2}\left[\frac{S(d^2-1)-(d+1)^2\Phi}{2(d-1)}\right]\right\}^{-1} \\
&= \frac{1}{K}\left\{\frac{b}{a^2-b^2}\cdot\frac{(d+1)}{2(d-1)}\left[S(d-1)-(d+1)\Phi\right]\right\}^{-1} \\
&= \frac{1}{K}\left\{\frac{1}{2(a-b)^2}\cdot\left[S(a-b)-(a+b)\Phi\right]\right\}^{-1} \\
&= \frac{2(a-b)^2}{K\left[S(a-b)-\sqrt{a^2-b^2}\frac{(a+\sqrt{a^2-b^2})^S-b^S}{(a+\sqrt{a^2-b^2})^S+b^S}\right]} \\
&= \frac{2a(1-\psi)^2}{K\left[S(1-\psi)-\sqrt{1-\psi^2}\frac{(1+\sqrt{1-\psi^2})^S-\psi^S}{(1+\sqrt{1-\psi^2})^S+\psi^S}\right]}.
\end{aligned}$$

C.3 Linear period effects, using expression (3)

Consider a basic staircase design with S unique treatment sequences. Assuming a linear time effect over the trial periods, the matrices encoding the time effects, $\mathbf{Z}_s$, $s = 1, \ldots, S$, are 2×2-dimensional matrices, with ones in the first column and the elements s and $s+1$ in the second column:

$$\mathbf{Z}_1 = \begin{pmatrix} 1 & 1 \\ 1 & 2 \end{pmatrix}, \quad \mathbf{Z}_2 = \begin{pmatrix} 1 & 2 \\ 1 & 3 \end{pmatrix}, \quad \cdots, \quad \mathbf{Z}_S = \begin{pmatrix} 1 & S \\ 1 & S+1 \end{pmatrix}$$

and so

$$\begin{aligned}
\sum_{s=1}^{S}\mathbf{Z}_s^\top\mathbf{V}_*^{-1}\mathbf{Z}_s &= \frac{b}{a^2-b^2}\begin{pmatrix} \sum_{s=1}^{S}2(d-1) & \sum_{s=1}^{S}(2s+1)(d-1) \\ \sum_{s=1}^{S}(2s+1)(d-1) & \sum_{s=1}^{S}\left[d+2s(s+1)(d-1)\right] \end{pmatrix} \\
&= \frac{b}{a^2-b^2}\begin{pmatrix} 2S(d-1) & S(S+2)(d-1) \\ S(S+2)(d-1) & Sd+\frac{2}{3}S(S+1)(S+2)(d-1) \end{pmatrix} \\
&= \frac{b}{a^2-b^2}\mathbf{Q}.
\end{aligned}$$

Then

$$\mathbf{Q}^{-1} = \begin{pmatrix} \frac{(2S(S+3)+7)d-2(S+2)(S+1)}{S^3(d-1)^2+2S(d+2)(d-1)} & \frac{-3(S+2)(d-1)}{S^3(d-1)^2+2S(d+2)(d-1)} \\ \frac{-3(S+2)(d-1)}{S^3(d-1)^2+2S(d+2)(d-1)} & \frac{6(d-1)}{S^3(d-1)^2+2S(d+2)(d-1)} \end{pmatrix}.$$

Now consider

$$\sum_{s=1}^{S}\mathbf{Z}_s\left(\sum_{s=1}^{S}\mathbf{Z}_s^\top\mathbf{V}_*^{-1}\mathbf{Z}_s\right)^{-1}\sum_{s=1}^{S}\mathbf{Z}_s^\top = \frac{a^2-b^2}{b}\sum_{s=1}^{S}\mathbf{Z}_s\mathbf{Q}^{-1}\sum_{s=1}^{S}\mathbf{Z}_s^\top.$$

Since

$$\sum_{s=1}^{S}\mathbf{Z}_s = \begin{pmatrix} \sum_{s=1}^{S}1 & \sum_{s=1}^{S}s \\ \sum_{s=1}^{S}1 & \sum_{s=1}^{S}(s+1) \end{pmatrix} = \begin{pmatrix} S & \frac{1}{2}S(S+1) \\ S & \frac{1}{2}S(S+1)+S \end{pmatrix},$$

then

$$\sum_{s=1}^{S}\mathbf{Z}_s\mathbf{Q}^{-1}\sum_{s=1}^{S}\mathbf{Z}_s^\top = \begin{pmatrix} \frac{(S^3+5S)d+S^3+S}{2((S^2+2)d-S^2+4)(d-1)} & \frac{S((S^2-1)d-S^2+7)}{2((S^2+2)d-S^2+4)(d-1)} \\ \frac{S((S^2-1)d-S^2+7)}{2((S^2+2)d-S^2+4)(d-1)} & \frac{(S^3+5S)d+S^3+S}{2((S^2+2)d-S^2+4)(d-1)} \end{pmatrix}$$

and so

$$\mathbf{X}^\top \mathbf{V}_*^{-1} \sum_{s=1}^{S} \mathbf{Z}_s \mathbf{Q}^{-1} \sum_{s=1}^{S} \mathbf{Z}_s^\top \mathbf{V}_*^{-1} \mathbf{X} = \frac{b}{a^2-b^2} \cdot \begin{pmatrix} -1 & d \end{pmatrix} \begin{pmatrix} \frac{(S^3+5S)d+S^3+S}{2((S^2+2)d-S^2+4)(d-1)} & \frac{S((S^2-1)d-S^2+7)}{2((S^2+2)d-S^2+4)(d-1)} \\ \frac{S((S^2-1)d-S^2+7)}{2((S^2+2)d-S^2+4)(d-1)} & \frac{(S^3+5S)d+S^3+S}{2((S^2+2)d-S^2+4)(d-1)} \end{pmatrix} \begin{pmatrix} -1 \\ d \end{pmatrix}$$

$$= \frac{b}{a^2-b^2} \cdot \frac{S((S^2+5)d^2 - 2d(S^2-4) + S^2 - 1)}{2d(S^2+2) - 2S^2 + 8}.$$

Then the variance of the treatment effect estimator is given by

$$\begin{aligned} var(\hat{\theta})_{SC(S,K,1,1),lin} &= \frac{1}{K} \left[S\mathbf{X}^\top \mathbf{V}_*^{-1} \mathbf{X} - \mathbf{X}^\top \mathbf{V}_*^{-1} \sum_{s=1}^{S} \mathbf{Z}_s \left(\sum_{s=1}^{S} \mathbf{Z}_s^\top \mathbf{V}_*^{-1} \mathbf{Z}_s \right)^{-1} \sum_{s=1}^{S} \mathbf{Z}_s^\top \mathbf{V}_*^{-1} \mathbf{X} \right]^{-1} \\ &= \frac{1}{K} \left\{ \frac{b}{a^2-b^2} \left[Sd - \frac{S((S^2+5)d^2 - 2d(S^2-4) + S^2 - 1)}{2d(S^2+2) - 2S^2 + 8} \right] \right\}^{-1} \\ &= \frac{1}{K} \left\{ \frac{b}{a^2-b^2} \cdot \frac{S(S^2-1)(d^2-1)}{2d(S^2+2) - 2S^2 + 8} \right\}^{-1} \\ &= \frac{2(S^2+2)a - 2(S^2-4)b}{KS(S^2-1)} \\ &= \frac{2a\left[(S^2+2) - (S^2-4)\psi\right]}{KS(S^2-1)}. \end{aligned}$$

C.4 Specific cases, $S \in \{2, 3, 4\}$

We present the analytical variance expressions for some specific cases: for $S = 2$, $S = 3$ and $S = 4$ unique treatment sequences.

C.4.1 Two unique treatment sequences ($S = 2$)

$$var(\hat{\theta})_{SC(2,K,1,1),cat} = \frac{2a}{K}$$

$$var(\hat{\theta})_{SC(2,K,1,1),lin} = \frac{2a}{K}$$

C.4.2 Three unique treatment sequences ($S = 3$)

$$var(\hat{\theta})_{SC(3,K,1,1),cat} = \frac{a\,(2-\psi)}{2K}$$

$$var(\hat{\theta})_{SC(3,K,1,1),lin} = \frac{a\,(11-5\psi)}{12K}$$

C.4.3 Four unique treatment sequences ($S = 4$)

$$var(\hat{\theta})_{SC(4,K,1,1),cat} = \frac{a\left(2-\psi^2\right)}{K(3+2\psi)}$$

$$var(\hat{\theta})_{SC(4,K,1,1),lin} = \frac{a\,(3-2\psi)}{5K}$$

References

Bowden R, Forbes AB, Kasza J. On the centrosymmetry of treatment effect estimators for stepped wedge and related cluster randomized trial designs. *Statistics and Probability Letters* 2021; 172: 109022.

da Fonseca CM and Petronilho J. Explicit inverses of some tridiagonal matrices. *Linear Algebra and its Applications* 2001; 325: 7-21.

Roebuck PA and Barnett S. A survey of Toeplitz and related matrices. *International Journal of Systems Science* 1978; 9(8): 921-934.

Tan LSL. Explicit inverse of tridiagonal matrix with applications in autoregressive modelling. *IMA Journal of Applied Mathematics* 2019; 84: 679-695.
